# RING‐Between‐RING‐Type E3 Ligase Ariadne‐Like Protein 8 Negatively Regulates Plant Virus Infection by Targeting a Viral Movement Protein

**DOI:** 10.1002/advs.202509942

**Published:** 2025-09-04

**Authors:** Wenli Li, Chenchen Zhong, Jiangning Duan, Changyi Zhan, Zhaolei Li, Xinyu Zhang, Dingliang Zhang, Deshui Liu, Zhiyan Wen, Xiaofei Zhao, Meng Yang, Dawei Li, Yongliang Zhang

**Affiliations:** ^1^ State Key Laboratory of Plant Environmental Resilience, College of Biological Sciences China Agricultural University Beijing 100193 China; ^2^ Beijing Life Science Academy Beijing 102200 China; ^3^ HouJi Laboratory in Shanxi Province, College of Agriculture Shanxi Agricultural University Taiyuan China

**Keywords:** barley stripe mosaic virus (BSMV), defense, RING‐between‐RING (RBR) E3 ligase Ariadne‐like protein 8 (ARI8), triple‐gene‐block (TGB) movement protein, ubiquitination

## Abstract

The ubiquitin–proteasome system is a highly conserved machinery that plays a crucial role in plant defense against viruses. However, the number of E3 ligases targeting viral proteins remains limited. Although RING‐between‐RING (RBR)‐type E3 ligases are evolutionarily conserved across organisms, their functions in plant responses to biotic stress remain largely unknown. Herein, it is found that the triple gene block 1 (TGB1) protein of the barley stripe mosaic virus (BSMV) undergoes ubiquitination during viral infection. Immunoprecipitation combined with mass spectrometry identified an RBR‐type E3 ligase that interacted with TGB1 in vivo and in vitro. The overexpression of Ariadne‐like protein 8 (ARI8) inhibits, whereas its knockout enhances, the local and systemic spread of BSMV. ARI8 mediated the ubiquitination of TGB1, and its Cys311 residue is required for the ARI8‐mediated degradation of TGB1 and inhibition of BSMV infection. In addition to BSMV, ARI8 negatively regulates infection by other TGB‐containing viruses, including potato virus X and beet necrotic yellow vein virus. Collectively, the findings identified a new E3 ligase that targets a plant viral protein and reveals a previously uncharacterized role for RBR‐type E3 ligases in plant responses to biotic stress, providing a potential molecular target for the development of antiviral strategies in plants.

## Graphical abstract

The plant RING‐between‐RING (RBR)‐type E3 ligase, Ariadne‐like protein 8 (ARI8), ubiquitinates and degrades a viral movement protein, restricting virus spread. ARI8 also inhibits other triple gene block 1 (TGB1)‐containing viruses, revealing a broad‐spectrum antiviral role. This study uncovers a previously unknown function of RBR‐type E3 ligases in plant immunity and offers a potential target for antiviral crop improvement.

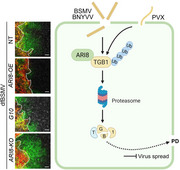

## 1 Introduction

During the long‐term coevolution of plants and viruses, plants have developed multilayered defense systems at the levels of DNA, RNA, proteins, hormones, and more as part of the ongoing arms race to counter viral attacks.^[^ ^1^, ^2^ ^]^ Ubiquitination, a highly conserved post‐translational modification mediated by the ubiquitin–proteasome system (UPS) in eukaryotes, plays a pivotal role in regulating plant growth, development, and responses to various biotic and abiotic stresses.^[^ ^3^, ^4^, ^5^, ^6^ ^]^ Increasing evidence suggests that the plant UPS is crucial during plant–virus interactions, as on the one hand, it acts as an important barrier of defense against viral invasion, and on the other hand, viruses have evolved mechanisms to hijack the UPS for their benefit.^[^ ^7^, ^8^, ^9^ ^]^

Upon viral invasion, plants employ the UPS to directly target virus‐encoded proteins for ubiquitination and subsequent degradation, restricting viral infection.^[^ ^10^ ^]^ Among these viral proteins, movement proteins (MPs) are the common targets of the UPS. For example, the MPs of tobacco mosaic virus (TMV),^[^ ^11^ ^]^ turnip yellow mosaic virus,^[^ ^12^ ^]^ potato leafroll virus,^[^ ^13^ ^]^ and beet necrotic yellow vein virus (BNYVV)^[^ ^14^ ^]^ undergo ubiquitination during viral infections. However, despite numerous reports on the UPS‐mediated degradation of viral MPs, the E3 ubiquitin ligases responsible for their ubiquitination remain largely unknown.

Ariadne‐like protein 8 (ARI8) belongs to the Ariadne‐like subclass of RING1‐between‐RING2 (RBR) E3 ubiquitin ligases, which are characterized by a C‐terminal Ariadne domain. In mammals, RBR‐type E3 ligases mediate ubiquitination through a three‐step mechanism. First, the RING1 domain binds to the ubiquitin–E2 conjugate. Second, ubiquitin is transferred to an active‐site cysteine (Cys) in the RING2 domain, and ubiquitin is transferred from RING2 to the substrate protein.^[^ ^15^, ^16^ ^]^ Although Ariadne family proteins (ARIs) are highly conserved and have been identified as putative E3 ligases across diverse organisms, including humans, *Drosophila*, mice, and *Arabidopsis thaliana*,^[^ ^15^, ^17^, ^18^, ^19^, ^20^, ^21^, ^22^ ^]^ their precise biological functions and the underlying mechanisms, particularly in plants, remain poorly understood. Studies on plant ARIs have primarily focused on their roles in plant development and responses against abiotic stresses. For example, the overexpression of ARI1 enhances aluminum tolerance in *Arabidopsis*,^[^ ^23^ ^]^ *At*ARI12 participates in ultraviolet B signaling by physically interacting with CONSTITUTIVE PHOTOMORPHOGENIC 1,^[^ ^24^, ^25^ ^]^ and ARI7 has been reported to function in reproductive development.^[^ ^26^ ^]^ However, the roles of ARI proteins in plant responses to biotic stress remain largely unknown. A recent study reported that the rice RBR‐type E3 ligase, RBRL, enhances antiviral immunity by ubiquitinating and degrading NOVEL INTERACTOR OF JAZ 3 (NINJA3), a repressor of jasmonic acid (JA) signaling, thereby activating JA‐dependent defense pathways.^[^ ^27^ ^]^ In contrast, the RBR‐type E3 ubiquitin ligase RING finger protein 217 of *N. benthamiana* degrades nucleotide‐binding, leucine‐rich repeat receptors required for cell death 4 (NRC4) to suppress plant immunity.^[^ ^28^ ^]^ Nonetheless, it has not yet been reported whether RBR‐type E3 ligases can directly ubiquitinate pathogen‐encoded proteins.

In this study, we used barley stripe mosaic virus (BSMV), a member of the genus *Hordeivirus* in the family *Virgaviridae*, and found that its MP, the triple gene block 1 (TGB1) protein, undergoes ubiquitination during viral infection. Through co‐immunoprecipitation (Co‐IP) and mass spectrometry analyses, we identified the RBR‐type E3 ligase ARI8 as an interacting partner of TGB1 that mediates its ubiquitination and subsequent degradation, thereby negatively regulating BSMV infection. In addition to BSMV, ARI8 interacts with TGB1 proteins encoded by other viruses to modulate their infections. Our findings reveal a previously uncharacterized role of RBR‐type E3 ligases in plant responses to biotic stress, while providing new insights into how RBR‐type E3 ligases contribute to the defense against viruses encoding TGB‐like MPs.

## 2 Results

### 2.1 TGB1 Protein of BSMV is Ubiquitinated During Virus Infection

**Figure 1:**
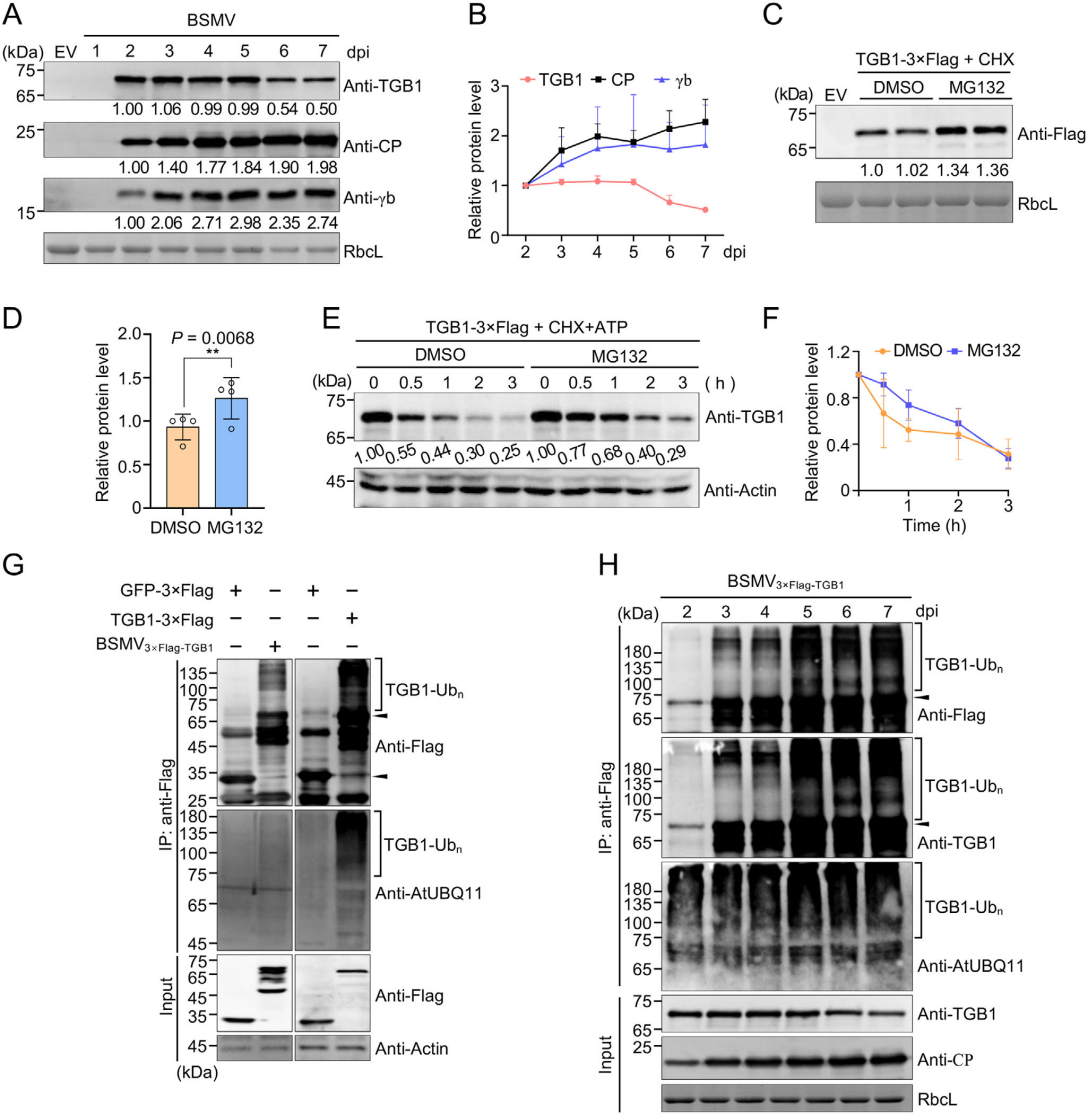
Triple gene block 1 (TGB1) protein is ubiquitinated and degraded by the 26S proteasome during barley stripe mosaic virus (BSMV) infection. A) Time‐course analysis of the accumulation levels of different BSMV‐encoded proteins during viral infection. The Rubisco large subunit (RbcL), detected using a stain‐free method, served as a loading control (bottom panel). B) Quantification of protein levels shown in (A). Data represent the mean ± standard deviation (SD) from three biological replicates. C) Immunoblot analysis showing increased accumulation of TGB1 protein following MG132 treatment. D) Quantification of the TGB1 levels shown in (C). Error bars represent the means ± SD (*n* = 4 biological replicates). Asterisks indicate statistically significant differences between the MG132‐treated and dimethyl sulfoxide (DMSO) control samples, as determined using a paired two‐tailed *t*‐test (**P <0.01). E) Cell‐free degradation assay assessing TGB1 stability. *Nicotiana benthamiana* leaves transiently expressing TGB1–3×Flag were harvested, and total protein extracts were incubated with 10 mM ATP and 0.5 mM cycloheximide (CHX) for the indicated times above the panel. TGB1 levels were examined by immunoblotting with anti‐TGB1 antibodies. Actin served as a loading control. F) Quantitative of TGB1 abundance as shown in (E). Data represent means ± SD from three biological replicates. G) Immunoblot analysis of TGB1 ubiquitination. Total proteins extracted from agroinfiltrated *N. benthamiana* leaves were immunoprecipitated using anti‐Flag agarose beads and analyzed by immunoblotting with the antibodies indicated on the right side of the panels. Solid and hollow arrowheads indicate the Flag‐tagged TGB1 (TGB1–3×Flag) and Flag‐tagged green fluorescent protein (GFP–3×Flag) bands, respectively. H) Time‐course analysis of TGB1 ubiquitination during BSMV infection. *Nicotiana benthamiana* leaves agroinoculated with BSMV_3×Flag‐TGB1_ were harvested at the indicated time points. Arrowheads indicate the TGB1 protein band.

During a time‐course analysis of the expression of BSMV‐encoded proteins in *Nicotiana benthamiana* leaves following BSMV infection, we observed that the accumulation levels of the coat protein (CP) and the pathogenicity determinant γb protein gradually increased over time. In contrast, the accumulation of the MP TGB1 initially increased but subsequently declined as the infection progressed (**Figure** **1**A,B), suggesting host‐mediated regulation of TGB1 stability during viral infection. Ubiquitination plays a crucial role in modulating protein stability.^[^ ^29^ ^]^ To investigate whether TGB1 is regulated by ubiquitination, FLAG‐tagged TGB1 was transiently expressed in *N. benthamiana* leaves, followed by an additional infiltration of the 26S proteasome inhibitor MG132 + cycloheximide (CHX) into the pre‐infiltrated leaves 12 h before sampling. MG132 treatment significantly increased TGB1 protein accumulation compared with that in the dimethyl sulfoxide (DMSO) + CHX control treatment (Figure 1C,D). Furthermore, the results of the in vitro cell degradation assay demonstrated that MG132 treatment markedly delayed TGB1 degradation (Figure 1E,F). These results suggest that TGB1 may undergo degradation through the 26S proteasome pathway.

To investigate whether TGB1 undergoes ubiquitination, we expressed BSMV_3×Flag–TGB1_, wherein Flag epitopes were fused to the N‐terminus of the TGB1 open reading frame (ORF) in the BSMV cDNA, or TGB1–3×Flag alone under the control of the 35S promoter in *N. benthamiana* using *Agrobacterium*‐mediated infiltration. Immunoblot analysis revealed the presence of high‐molecular‐weight smear bands above the TGB1 protein band in the context of BSMV infection or expression of the TGB1 protein alone. In contrast, no corresponding high‐molecular‐weight smear bands were observed in the control samples expressing FLAG‐tagged green fluorescent protein (GFP–3×Flag) (Figure 1G). Time‐course analysis of TGB1 ubiquitination during BSMV infection consistently showed that TGB1 ubiquitination increased over time (Figure 1H), which correlated with a gradual decline in TGB1 protein accumulation during the later stages of BSMV infection (Figure 1A). Collectively, these results demonstrated that TGB1 undergoes ubiquitination during BSMV infection, likely contributing to its decreased accumulation during the later stages of viral infection.

### 2.2 ARI8 Interacts with TGB1 Both in vivo and in vitro

To identify the E3 ligase responsible for ubiquitinating BSMV‐encoded TGB1, we used TGB1 protein as bait and transiently expressed 3×FLAG‐tagged TGB1 (TGB1–3×Flag) in *N. benthamiana* leaves. Immunoprecipitation (IP) was then performed to enrich potential TGB1‐interacting proteins, followed by liquid chromatography–tandem mass spectrometry analysis. The results revealed that ARI8 ranked the highest among the candidate E3 ligases interacting with TGB1 (Table S1, Supporting Information). ARI8 belongs to the RBR domain‐containing E3 ubiquitin ligases^[^ ^30^ ^]^ and is highly conserved across monocots and dicots (Figure S1, Supporting Information). The predicted three‐dimensional structures of ARI8 orthologs in humans, yeast, and plants also exhibited high similarity (Figure S2, Supporting Information), suggesting the functional conservation of ARI8 across kingdoms. Therefore, we focused on the subsequent studies on ARI8. A search of the *N. benthamiana* genome database of the Solanaceae (Sol) Genomics Network (https://solgenomics.net/) helped identify two paralogs of *N. benthamiana* *ARI8*: Niben101Scf03512g02004.1 and Niben101Scf08947g02007.1. Sequence alignment analysis of the two *N. benthamiana* *ARI8* paralogs, Niben101Scf03512g02004.1, and Niben101Scf08947g02007.1, revealed high nucleotide level (97.36%) and amino acid level (97.47%) (Figure S3A, Supporting Information). Structural modeling using AlphaFold3 further showed that the proteins encoded by these two paralogs shared a highly similar structure (Figure S3B, Supporting Information), suggesting a strong functional similarity between them. We successfully cloned *NbARI8* (Niben101Scf03512g02004.1) from *N. benthamiana* cDNA and used it for subsequent studies. Unless otherwise specified, in this study, ARI8 refers to ARI8 from *N. benthamiana*.

**Figure 2:**
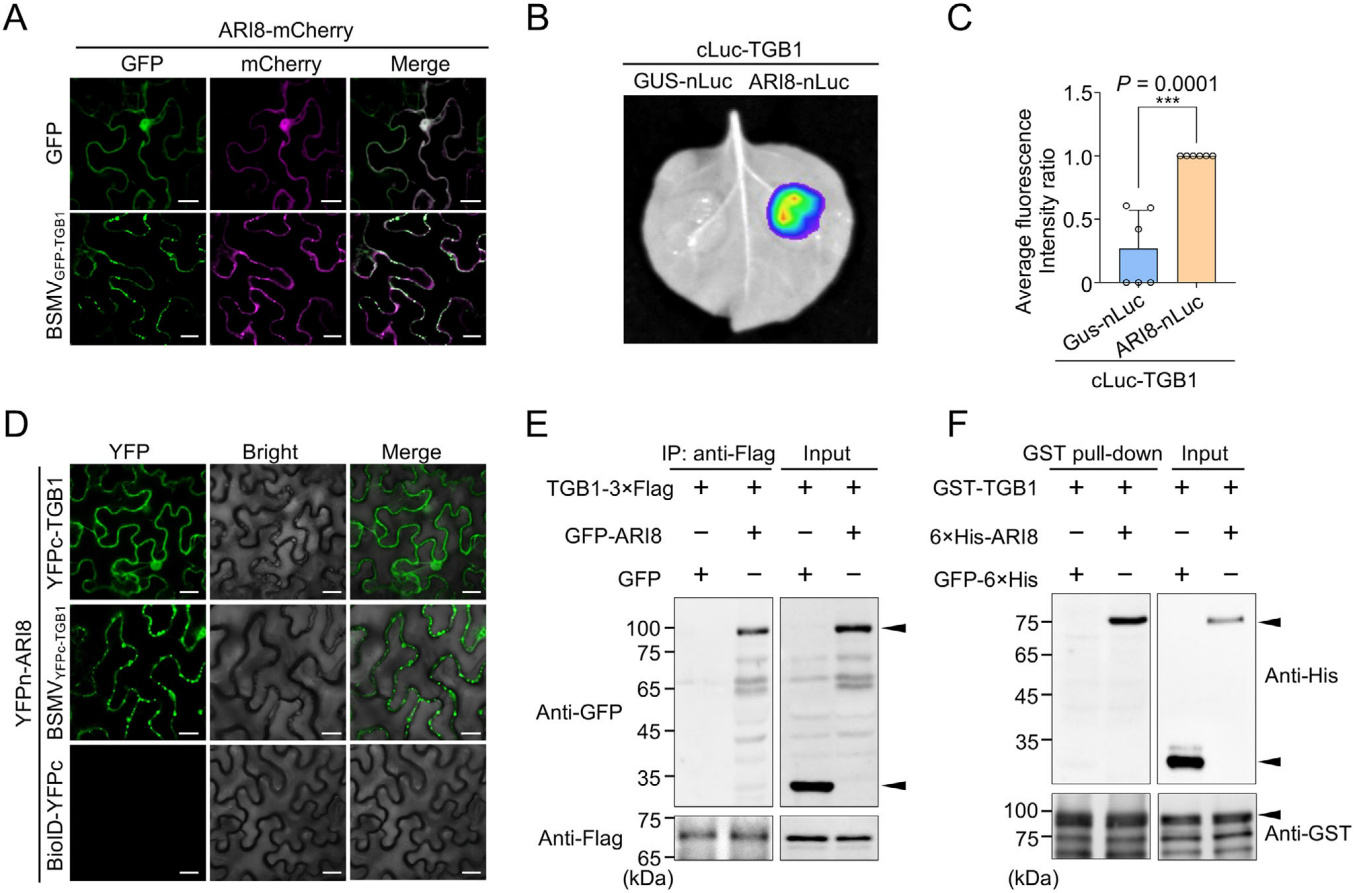
Ariadne‐like protein 8 (ARI8) interacts with the triple gene block 1 (TGB1) protein in vivo and in vitro. A) Subcellular co‐localization analysis of ARI8 and TGB1 in *Nicotiana benthamiana* leaves. Scale bars represent 20 µm. B) Luciferase complementation imaging (LCI) assay to test the interaction between ARI8 and TGB1. cLuc–TGB1 was co‐expressed with ARI8–nLuc or GUS–nLuc (negative control) in different regions of the same *N. benthamiana* leaves. Luminescence signals were recorded at 48 h post‐infiltration (hpi). C) Quantitative analysis of luminescence from (B), normalized to the average fluorescence intensity measured using ImageJ software. Data represent means ± standard deviation from six biologically independent plants. Statistical significance was assessed using an unpaired two‐tailed *t*‐test (***P <0.0001). D) Bimolecular fluorescence complementation (BiFC) assay showing the interaction between ARI8 and TGB1. ARI8 fused with the N‐terminal fragment of yellow fluorescent protein (YFPn–ARI8) was co‐expressed with TGB1 fused with the C‐terminal fragment of YFP (YFPc–TGB1) or the barley stripe mosaic virus (BSMV)‐derived TGB1 fused with YFPc (BSMV_YFPc–TGB1_) in *N. benthamiana* leaves. Scale bars represent 20 µm. The combination of YFPn–ARI8 and BioID–YFPc served as a negative control. E) Co‐immunoprecipitation (Co‐IP) assay demonstrating the interaction between ARI8 and TGB1. Total proteins were extracted from *N. benthamiana* leaves at 48 hpi and immunoprecipitated using anti‐Flag beads. Input and immunoprecipitated (IP) samples were analyzed by immunoblotting with anti‐green fluorescent protein (GFP) and anti‐Flag antibodies. F) Glutathione S‐transferase (GST) pull‐down assay confirming the direct interaction between ARI8 and TGB1 in vitro.

To examine whether ARI8 interacts with TGB1, we analyzed the subcellular localization of ARI8 using confocal microscopy. In the absence of BSMV infection, ARI8 localizes to both the nucleus and cytoplasm. However, during BSMV infection, GFP‐tagged TGB1 was used to mark the plasmodesmata (PD),^[^ ^31^ ^]^ revealing that ARI8 was redistributed and co‐localized with GFP–TGB1 in the PD (**Figure** **2**A; Figure S4, Supporting Information). The results of the immunoblot analysis confirmed protein expression in the co‐subcellular localization assay (Figure S5A, Supporting Information). These results suggest that ARI8 is likely involved in BSMV infection through its interaction with TGB1. Luciferase complementation imaging (LCI) was performed to validate the interaction between ARI8 and TGB1. The combination of cLuc–TGB1 and ARI8–nLuc produced a strong luciferase signal, whereas the control groups expressing the cLuc–TGB1 and GUS–nLuc recombinant proteins did not exhibit a luciferase signal (Figure 2B,C). Moreover, bimolecular fluorescence complementation (BiFC) assays revealed that the yellow fluorescent protein (YFP) signal was reconstituted when the N‐terminal fragment of YFP (YFPn)‐fused ARI8 was co‐expressed with the C‐terminal fragment of YFP (YFPc)‐fused TGB1 (Figure 2D). Additionally, when YFPc was directly fused to the N‐terminus of the TGB1 ORF within the BSMV infectious cDNA to yield BSMV_YFPc–TGB1_, co‐expression with YFPn‐ARI8 and BSMV_YFPc–TGB1_ generated YFP fluorescence (Figure 2D), similar to the PD localization of ARI8 observed during BSMV infection (Figure 2A; Figure S4, Supporting Information). Immunoblot analysis confirmed the protein expression in the BiFC assay (Figure S5B, Supporting Information). Co‐IP analysis revealed that TGB1 co‐precipitated with ARI8, but not with the GFP control (Figure 2E). Furthermore, an in vitro glutathione S‐transferase (GST) pull‐down assay showed that GST‐tagged TGB1 specifically bound to ARI8, but not to GFP (Figure 2F). Collectively, these results demonstrated that ARI8 interacts with TGB1 both in vivo and in vitro.

### 2.3 ARI8 Negatively Regulates BSMV Infection in the Host

To investigate the role of ARI8 in BSMV infection, we transiently expressed ARI8 and assessed its effects on BSMV accumulation. The results of the immunoblot analysis revealed that the expression of ARI8 significantly suppressed the accumulation of BSMV (Figure S6A,B, Supporting Information). In contrast, silencing the expression of ARI8 using tobacco rattle virus‐induced gene silencing enhanced the accumulation of BSMV (Figure S6C–E, Supporting Information). To further elucidate the role of ARI8 in BSMV infection, we generated transgenic *N. benthamiana* plants stably overexpressing ARI8 (*ARI8‐OE*). Phenotypic observations revealed that *ARI8‐OE* transgenic plants exhibited stunted growth compared with that observed in non‐transgenic (NT) plants (Figure S7A,B, Supporting Information). To facilitate the visualization of BSMV infection in *N. benthamiana*, we employed the split superfolder GFP (sfGFP) system.^[^ ^32^ ^]^ In this system, sfGFP1–10 (the 1st to 10th β‐strands of sfGFP) was stably transformed into *N. benthamiana*, resulting in the *G10* line,^[^ ^33^ ^]^ whereas the 11th β‐strand of sfGFP (sfGFP11) was fused to the C‐terminus of the γb ORF within the BSMV infectious cDNA to generate BSMV_γb–sfGFP11_. When BSMV_γb–sfGFP11_ was introduced into *G10* plants via agroinfiltration, sfGFP1–10 and sfGFP11 assembled into an intact sfGFP protein, enabling BSMV infection to be visualized under ultraviolet light. Additionally, we generated ARI8 knockout (*ARI8‐KO*) transgenic plants in a *G10* background. Both ARI8 paralogs, Niben101Scf03512g02004.1, and Niben101Scf08947g02007.1, were simultaneously knocked out in the *ARI8‐KO* plants. Phenotypic observations revealed that *ARI8‐KO* plants exhibited no apparent differences from the control *G10* plants (Figure S7C–E, Supporting Information).

**Figure 3:**
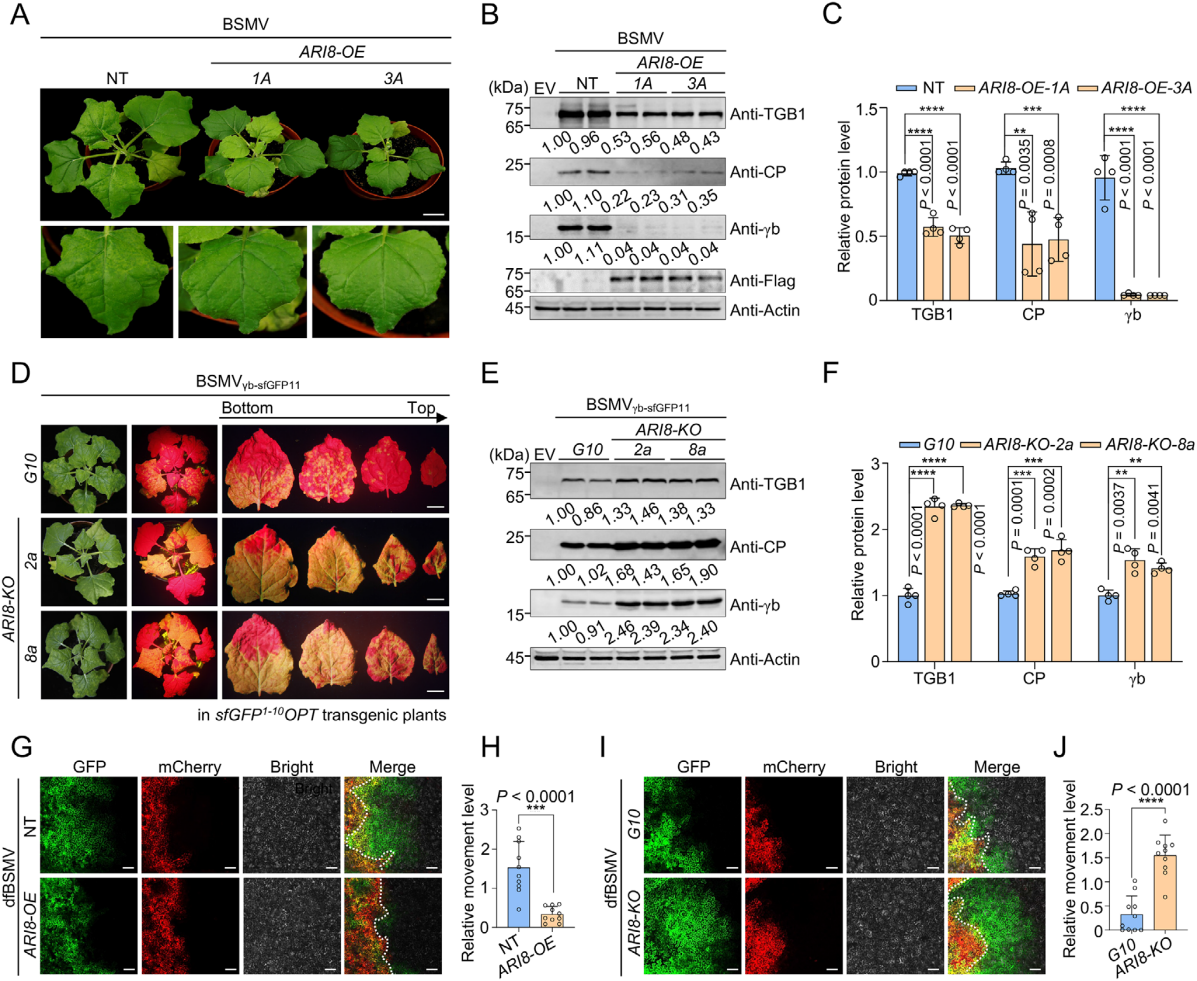
Ariadne‐like protein 8 (ARI8) negatively regulates barley stripe mosaic virus (BSMV) infection. A) Symptoms of BSMV infection in the leaves of non‐transgenic (NT) and wild‐type ARI8 overexpressing (*ARI8‐OE*) *Nicotiana benthamiana* plants at 10 days post‐inoculation (dpi). Enlarged views of systemically infected leaves are shown below. Scale bar represents 3.5 cm. B) Immunoblot analysis of viral protein accumulation in the systemic leaves shown in (A). Actin was used as a loading control. C) Quantification of viral protein levels in (B). D) Symptoms of ARI8 knockout (*ARI8‐KO*) *N. benthamiana* plants inoculated with the construct containing the 11th β‐strand of superfolder green fluorescent protein (sfGFP11) fused to the C‐terminus of the γb protein of BSMV (BSMV_γb‐sfGFP11_) at 9 dpi. *Nicotiana benthamiana* plants expressing the optimized superfolder GFP1–10 (sfGFP1–10^OPT^, *G10*) were infiltrated with the BSMV_γb–sfGFP11_ construct at a final optical density at 600 nm (OD_600_) of 0.1 for each component. GFP fluorescence was visualized under ultraviolet (UV) light equipped with a yellow filter. *G10* transgenic *N. benthamiana* served as the control. Scale bar, 1.5 cm. E) Immunoblot analysis of viral protein accumulation in systemic leaves from (D). F) Quantification of viral protein levels in (E). For panels (C) and F), error bars represent mean ± standard deviation (SD) (*n* = 4 biological repeats). Asterisks indicate statistically significant differences determined using an unpaired two‐tailed *t*‐test (**P <0.01, ***P <0.001, and ****P <0.0001). All experiments (A–F) were repeated at least twice, and representative results are shown. G) Confocal microscopy analysis of cell‐to‐cell movement of BSMV using the dfBSMV reporter system^[^ ^34^ ^]^ in *ARI8‐OE* plants. The NT plant served as a control. H) Quantification of BSMV movement in (G). I) Analyses of BSMV cell‐to‐cell movement using the dfBSMV reporter system in *ARI8‐KO* plants. *G10* plants served as the control. J) Quantification of BSMV cell‐to‐cell movement in (I). Representative images in (G) and (I) were captured at 62 and 58 h post‐infiltration (hpi), respectively. Scale bars represent 100 µm. The left side of the white dashed line marks the primary agroinfiltration zone, whereas the right side indicates areas with GFP signal spread beyond the infiltration region, reflecting the movement of the virus. Scale bars represent 100 µm. The Y‐axis shows the ratio of GFP‐positive area to the corresponding red fluorescent area. Data represent means ± SD (*n* = 10 biologically independent plants). Statistical significance was assessed using an unpaired two‐tailed Student's *t*‐test (***P <0.001, and ****P <0.0001).

To evaluate the effects of ARI8 overexpression on BSMV infection, BSMV was inoculated into NT and *ARI8‐OE* plants through agroinfiltration. At 10 days post‐infiltration (dpi), BSMV‐induced symptoms were markedly attenuated in the systemic leaves of *ARI8‐OE* plants compared to those in NT plants (**Figure** **3**A). Immunoblot analysis revealed that BSMV accumulation was significantly reduced in the systemic leaves of *ARI8‐OE* plants compared with that in the systemic leaves of NT plants (Figure 3B,C). Moreover, *Agrobacterium* mixtures carrying BSMV_γb–sfGFP11_ were infiltrated into both *G10* and *ARI8‐KO* plants. At 9 dpi, BSMV infection was notably stronger in *ARI8‐KO* plants than in the control *G10* plants, as evidenced by the higher GFP signal intensity observed in *ARI8‐KO* plants (Figure 3D). Immunoblot analysis further confirmed that BSMV accumulation in the systemic leaves of *ARI8‐KO* plants was significantly higher than that in the leaves of the *G10* control plants (Figure 3E,F). These results indicated that ARI8 negatively regulates BSMV infection. We cloned an ortholog of *N. benthamiana* ARI8 from barley (*Hordeum vulgare*; *HvARI8*). BiFC and Co‐IP assays confirmed that *Hv*ARI8 also interacted with BSMV‐encoded TGB1 (Figures S8A,B; Figure S5D, Supporting Information). Moreover, the transient overexpression of *Hv*ARI8 suppressed BSMV infection (Figure S8C, Supporting Information), suggesting a conserved antiviral role for ARI8 against BSMV across different plant species.

We further investigated how ARI8 negatively regulates BSMV infection, considering that ARI8 localizes to the PD during BSMV infection and interacts with TGB1, thereby suggesting that ARI8 may inhibit BSMV infection by affecting viral movement. To test this hypothesis, we infiltrated *Agrobacterium* harboring movement‐deficient BSMV (RNAα + RNAγ) into *ARI8‐OE* or *ARI8‐KO* plants to assess viral replication. Immunoblot analysis revealed that neither *ARI8* overexpression nor knockout had a pronounced effect on BSMV replication, thus excluding the role of ARI8 in BSMV replication (Figure S6F–I, Supporting Information). Furthermore, we used a previously established dual‐fluorescence reporter system, dfBSMV, to monitor the cell‐to‐cell movement of BSMV, wherein red fluorescence indicates the infiltration site, and green fluorescence outside the red region represents the cell‐to‐cell movement of the virus.^[^ ^34^ ^]^ When dfBSMV was inoculated into *ARI8‐OE* plants, viral movement was suppressed (Figure 3G,H). Conversely, BSMV exhibited enhanced movement in *ARI8‐KO* plants compared with that in *G10* plants (Figure 3I,J). Collectively, these findings suggest that ARI8 negatively regulates the infection of BSMV primarily by inhibiting viral movement.

### 2.4 E3 Ligase ARI8 Mediates the Ubiquitination and Degradation of TGB1

**Figure 4:**
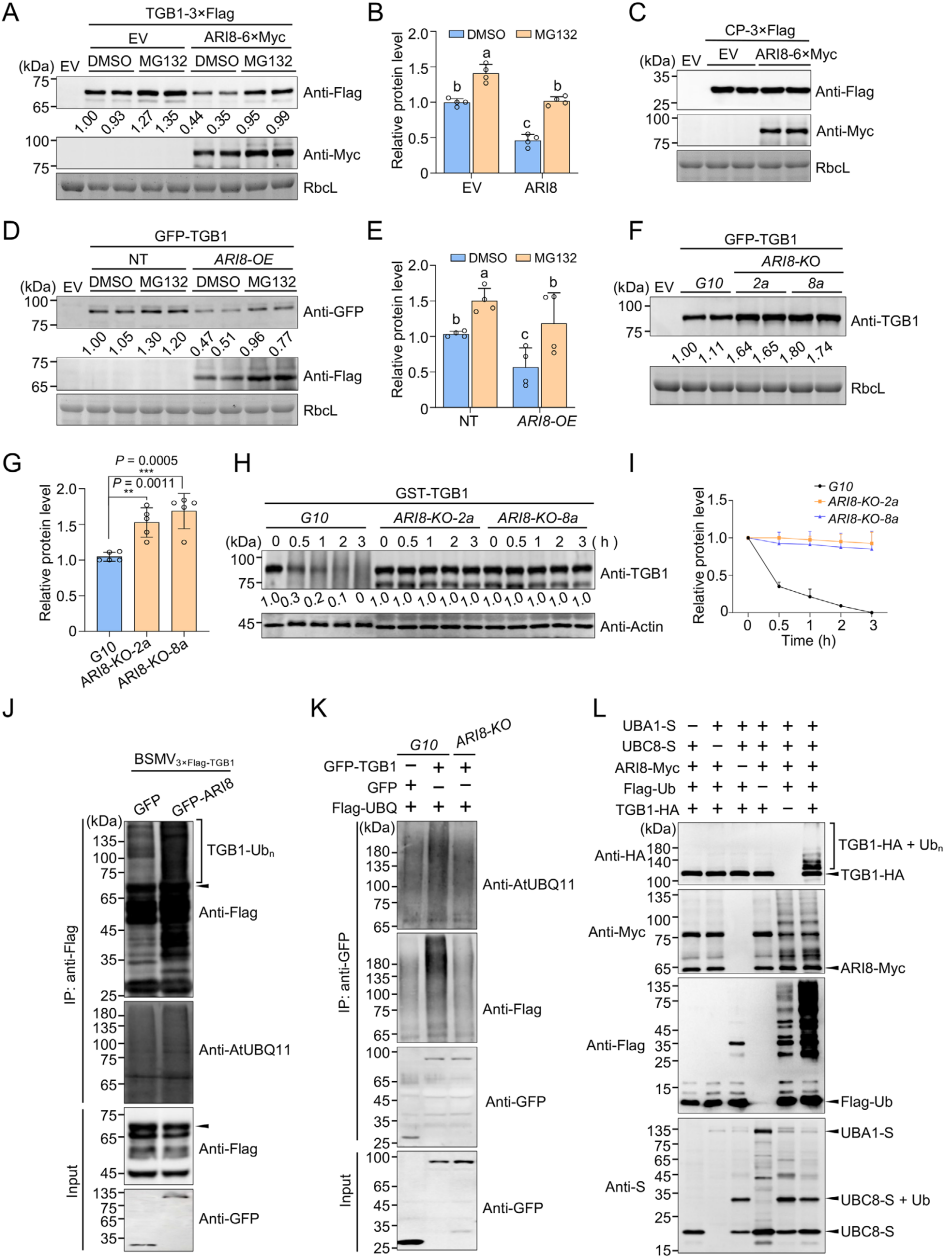
Ariadne‐like protein 8 (ARI8) mediates the ubiquitination and 26S proteasome‐dependent degradation of the triple gene block 1 (TGB1) protein. A) Overexpression of ARI8 reduces TGB1 protein accumulation, a process that can be mitigated by MG132 treatment. TGB1–3×Flag was co‐expressed with ARI8 or empty vector (EV, control) in *Nicotiana benthamiana* leaves. At 36 h post‐infiltration (hpi), 100 µM MG132 or an equivalent volume of dimethyl sulfoxide (DMSO) (control) was infiltrated into the pre‐infiltrated leaf regions. Approximately 12 h later, the infiltrated leaves were harvested for immunoblot analysis. RbcL was used as a loading control. B) Quantification of TGB1 protein levels in (A). C) Overexpression of ARI8 has no pronounced effect on the accumulation of the coat protein (CP) of the barley stripe mosaic virus (BSMV). D) Immunoblot analysis of TGB1 levels in non‐transgenic (NT) and *ARI8‐OE* plants. E) Quantification of the TGB1 protein levels in (D). In (B) and (E), error bars represent the mean ± standard deviation (SD) (*n* = 4 biological repeats). Different letters indicate statistically significant differences among groups, as determined by Duncan's multiple range test (*P* <0.05). F) Knockout (KO) of *ARI8* enhances TGB1 accumulation. *Agrobacterium* carrying green fluorescent protein (GFP)‐tagged TGB1 (GFP–TGB1) was infiltrated into the leaves of *ARI8‐KO* plants. Leaf tissues were collected at 2 days post‐inoculation (dpi) and subjected to immunoblot analysis. *G10* plants served as controls. G) Quantification of the TGB1 levels shown in (F). Error bars represent mean ± SD (*n* = 4 biological repeats). Asterisks indicate statistically significant differences based on an unpaired two‐tailed *t*‐test (**P <0.01, and ***P <0.001). H) Cell‐free degradation assay assessing TGB1 stability. Purified recombinant glutathione S‐transferase (GST)–TGB1 protein was incubated with an equal amount of leaf extracts from *G10* (control) or *ARI8‐KO* plants in the presence of 10 mM ATP and 0.5 mM cycloheximide (CHX) for the indicated periods. Protein levels were examined by immunoblotting with anti‐TGB1 antibodies. Actin was used as a loading control. I) Quantification of TGB1 abundance in (H). Data represent the mean ± SD from four biological replicates. J) Overexpression of ARI8 enhances TGB1 ubiquitination during BSMV infection. Flag‐tagged TGB1 expressed from BSMV was co‐expressed with GFP (control) or GFP‐ARI8 in *N. benthamiana* leaves. At 36 hpi, MG132 or DMSO was infiltrated into the pre‐infiltrated leaf regions. At 48 hpi, total proteins were extracted and immunoprecipitated using anti‐Flag agarose beads, followed by immunoblot analysis with the indicated antibodies. TGB1–Ub_n_ indicates polyubiquitinated TGB1 protein. Arrowheads indicate 3×Flag‐TGB1 protein band. K) Knockout of *ARI8* reduces TGB1 ubiquitination level. GFP–TGB1 and Flag–UBQ were co‐expressed in *G10* or *ARI8‐KO* plants through *Agrobacterium*‐mediated infiltration. Approximately 48 h later, total proteins were extracted, immunoprecipitated using anti‐GFP agarose beads, and analyzed by immunoblotting with the antibodies indicated on the right. L) ARI8 ubiquitinates TGB1 in vitro. Immunoblot analysis was performed on bacterial lysates from *E. coli* expressing FLAG‐Ub, AtUBA1(E1)‐S, AtUBC8(E2)‐S, ARI8‐MYC, and TGB1–HA, or strains lacking one of these components. Anti‐HA antibodies were used to detect TGB1 ubiquitination; anti‐Myc to detect the E3 ligase activity of ARI8, and anti‐S to validate the expression of E1 and E2 enzymes. All experiments were independently repeated at least twice, and similar results were obtained.

To further characterize the functional relationship between ARI8 and TGB1, we co‐expressed TGB1 and ARI8 in *N. benthamiana* leaves using agroinfiltration. The results revealed that the overexpression of ARI8 reduced the accumulation of TGB1 protein compared with that in the EV‐expressing control plants, whereas the treatment with the 26S proteasome inhibitor MG132 alleviated the ARI8‐induced reduction in TGB1 accumulation (**Figure** **4**A,B). Likewise, transient overexpression of HvARI8 reduced the accumulation of BSMV TGB1 protein, a process that could be mitigated by MG132 treatment (Figure S8D, Supporting Information). As a control, ARI8 overexpression had no pronounced effect on the protein levels of CP, another BSMV‐encoded protein (Figure 4C). Furthermore, GFP‐tagged TGB1 was expressed in both NT and *ARI8‐OE* plants via agroinfiltration, followed by the additional infiltration of MG132 and CHX into pre‐infiltrated leaves 12 h before sampling. Immunoblot analysis showed that MG132 treatment significantly increased TGB1 protein accumulation (Figure 4D), consistent with the results shown in Figure 1C. In contrast, ARI8 overexpression significantly reduced TGB1 protein levels, which were mitigated by the MG132 treatment (Figure 4D,E). Furthermore, GFP–TGB1 was expressed in both *ARI8‐KO* and *G10 N. benthamiana* plants via agroinfiltration. Immunoblot analysis revealed that TGB1 protein accumulation was significantly higher in *ARI8‐KO* plants than in the *G10* control plants (Figure 4F,G). The in vitro cell‐free degradation assay further demonstrated that the TGB1 protein underwent noticeable degradation over time in the *G10* control plants, whereas its degradation was significantly suppressed in *ARI8‐KO* plants (Figure 4H,I). These results suggest that ARI8 likely promote the degradation of TGB1 through the 26S proteasome pathway.

To further examine whether ARI8 mediates the ubiquitination of TGB1, BSMV_3×Flag–TGB1_ was co‐expressed with GFP–ARI8 in *N. benthamiana* leaves and the effects of ARI8 expression on TGB1 ubiquitination levels were analyzed. Immunoblot analysis of TGB1 protein enriched by IP revealed that compared to the GFP control, overexpression of ARI8 enhanced TGB1 ubiquitination (Figure 4J). Additionally, when GFP–TGB1 was expressed in *G10* plants, immunoblot analysis of immunoprecipitated TGB1 revealed the ubiquitination of TGB1 (Figure 4K), consistent with the results shown in Figure 1G. However, knockout of *ARI8* significantly reduced TGB1 ubiquitination levels compared to *G10* plants (Figure 4K). These results indicated that ARI8 promotes the ubiquitination of TGB1 in vivo, leading to its degradation via the 26S proteasome. To determine whether ARI8 could directly ubiquitinate TGB1, an in vitro ubiquitination assay was performed using a bacterial ubiquitination system.^[^ ^35^ ^]^ In this system, AtUBA1 (E1), AtUBC8 (E2), ARI8‐Myc (E3), FLAG‐tagged ubiquitin (FLAG‐Ub), and the substrate MBP‐TGB1‐HA were co‐expressed in *Escherichia coli*. Immunoblot analysis showed that ARI8 possesses E3 ubiquitin ligase activity and that TGB1 is ubiquitinated by ARI8, as evidenced by the appearance of high‐molecular‐weight smear bands above the TGB1 protein band (Figure 4L). Collectively, these results demonstrated that the E3 ubiquitin ligase ARI8 directly ubiquitinates TGB1 and mediates its degradation through the 26S proteasome.

### 2.5 E3 Ligase Activity of ARI8 is Required for Antiviral Defense

**Figure 5:**
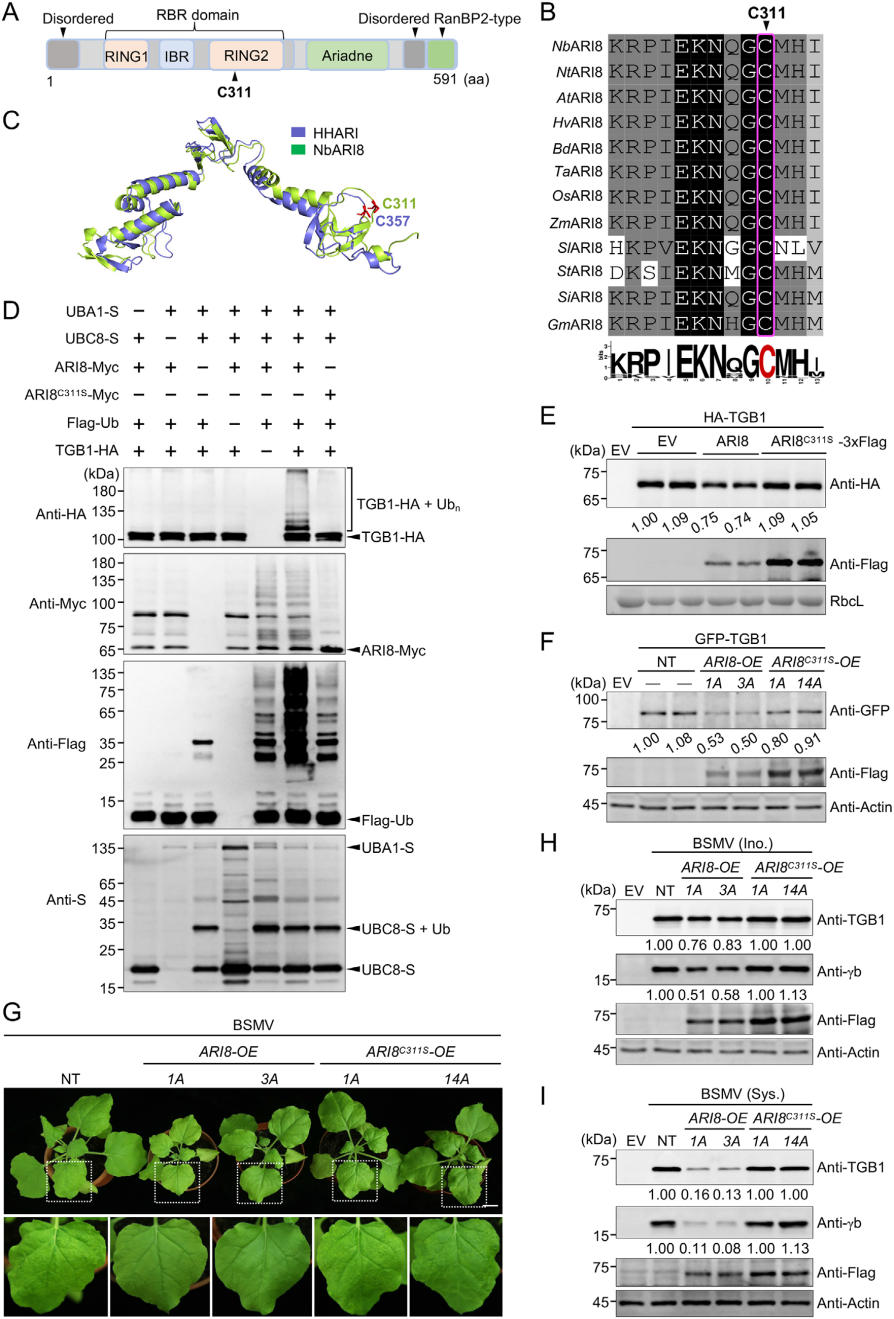
E3 ligase activity of Ariadne‐like protein 8 (ARI8) is required for defense against barley stripe mosaic virus (BSMV) infection. A) Schematic diagram showing the functional domains of ARI8. B) Amino acid sequence alignment reveals that the Cys311 residue is highly conserved across diverse plant species. The sequence logos shown below were generated using the online WebLogo tool (https://weblogo.berkeley.edu/logo.cgi). C) Structural modeling of ARI8 reveals that the Cys311 residue, which localizes in the RING2 domain, corresponds to Cys357 of human homolog of Ariadne (HHARI). D) The ARI8^C311S^ mutant protein fails to ubiquitinate the triple gene block 1 (TGB1) protein in vitro. Immunoblot analysis was performed using bacterial lysates as described in Figure 4L. E) Transient overexpression of the ARI8^C311S^ mutant protein does not reduce TGB1 protein accumulation. F) Immunoblot analysis of TGB1 accumulation in non‐transgenic (NT), wild‐type ARI8 overexpressing (*ARI8‐OE*), and mutant ARI8 overexpressing (*ARI8^C311S^‐OE*) *Nicotiana benthamiana* plants. G) Symptoms of BSMV infection in NT, *ARI8‐OE*, and *ARI8^C311S^*‐*OE N. benthamiana* plants. Photographs were taken at 7 days post‐inoculation (dpi). Systemically infected leaves are enlarged and shown below. Scale bar, 3.5 cm. H, I) Immunoblot analysis of BSMV accumulation in inoculated (H) and systemic leaves (I). Actin served as a loading control. All experiments were independently repeated at least twice, and similar results were obtained.

To further characterize the ARI8‐mediated degradation of TGB1, we analyzed the key catalytic site of ARI8. The RING2 domain of ARI8 receives ubiquitin molecules transferred from the RING1 domain and subsequently transfers them to substrate proteins (**Figure** **5**A), thereby exerting E3 ligase activity.^[^ ^15^, ^16^ ^]^ ARI8 is highly conserved in humans, yeast, and plants (Figure 5B; Figure S2, Supporting Information), and a mutation at the catalytic site (Cys357) in the RING2 domain of ARI8 ortholog, human homolog of Ariadne (HHARI), abolishes its ability to transfer ubiquitin, thereby disrupting its enzymatic activity.^[^ ^15^, ^16^ ^]^ The results of sequence alignment and protein structure prediction revealed that the Cys311 residue in ARI8 corresponds to Cys357 in HHARI and is highly conserved across various plant species (Figure 5B,C), suggesting that a mutation at Cys311 may affect ARI8 enzymatic activity in plants. To test this hypothesis, we substituted the Cys311 residue with a serine residue (C311S) and performed an in vitro ubiquitination assay. The results demonstrate that the E3 ligase activity of ARI8^C311S^ was significantly impaired, as evidenced by its markedly reduced ability to ubiquitinate the substrate TGB1 (Figure 5D). Moreover, the transient overexpression of the ARI8^C311S^ mutant protein failed to reduce TGB1 accumulation, unlike that caused by the overexpression of wild‐type ARI8 (Figure 5E). Split‐luciferase (split‐LUC) (Figure S9A, Supporting Information) and BiFC assays (Figure S9B and S5E, Supporting Information) confirmed that ARI8^C311S^ retained a strong interaction with TGB1, ruling out the possibility that its reduced impact was because of the weakened binding of ARI8^C311S^ to TGB1. These results indicate that the E3 ligase activity of ARI8 is essential for reducing TGB1 accumulation levels.

To further investigate the effects of the C311S mutation on ARI8 function, we generated transgenic *N. benthamiana* plants overexpressing ARI8^C311S^ (*ARI8^C311S^*‐*OE*). Phenotypic analysis revealed that *ARI8^C311S^‐OE* plants exhibited no apparent differences in growth compared with NT plants, a phenotype distinct from that of *ARI8‐OE* plants (Figure S7F,G, Supporting Information), likely because of the disruption of the E3 ligase activity of ARI8. Moreover, the expression of TGB1 in these three types of *N. benthamiana* plants revealed that TGB1 accumulation was lower in *ARI8‐OE* plants than in NT plants. However, TGB1 accumulation in *ARI8^C311S^‐OE* plants was similar to that in the NT plants (Figure 5F). These findings are consistent with the results shown in Figure 5E, further indicating that the inactivation of ARI8 E3 ligase activity abolishes the ARI8‐mediated degradation of TGB1.

Furthermore, we inoculated the wild‐type (WT), *ARI8‐OE*, and *ARI8^C311S^‐OE* plants with BSMV through agroinfiltration. The results showed that BSMV‐induced symptoms in the systemic leaves of *ARI8‐OE* plants were substantially alleviated compared to those in NT plants. In contrast, BSMV‐induced symptoms in *ARI8^C311S^‐OE* plants were similar to those observed in NT plants (Figure 5G). Immunoblot analysis of the local (Ino.) and systemic (Sys.) leaves showed that BSMV accumulation was significantly reduced in *ARI8‐OE* plants compared to NT plants. However, BSMV accumulation in *ARI8^C311S^‐OE* plants was comparable to that in NT plants (Figure 5H,I). These results indicate that the loss of ARI8 E3 ligase activity abolished its inhibitory effect on BSMV infection. Collectively, these results demonstrated that the E3 ligase activity of ARI8 is indispensable for defense against BSMV infections.

### 2.6 ARI8 Functions in Regulating the Infections of Other TGB‐Harboring Plant Viruses

**Figure 6:**
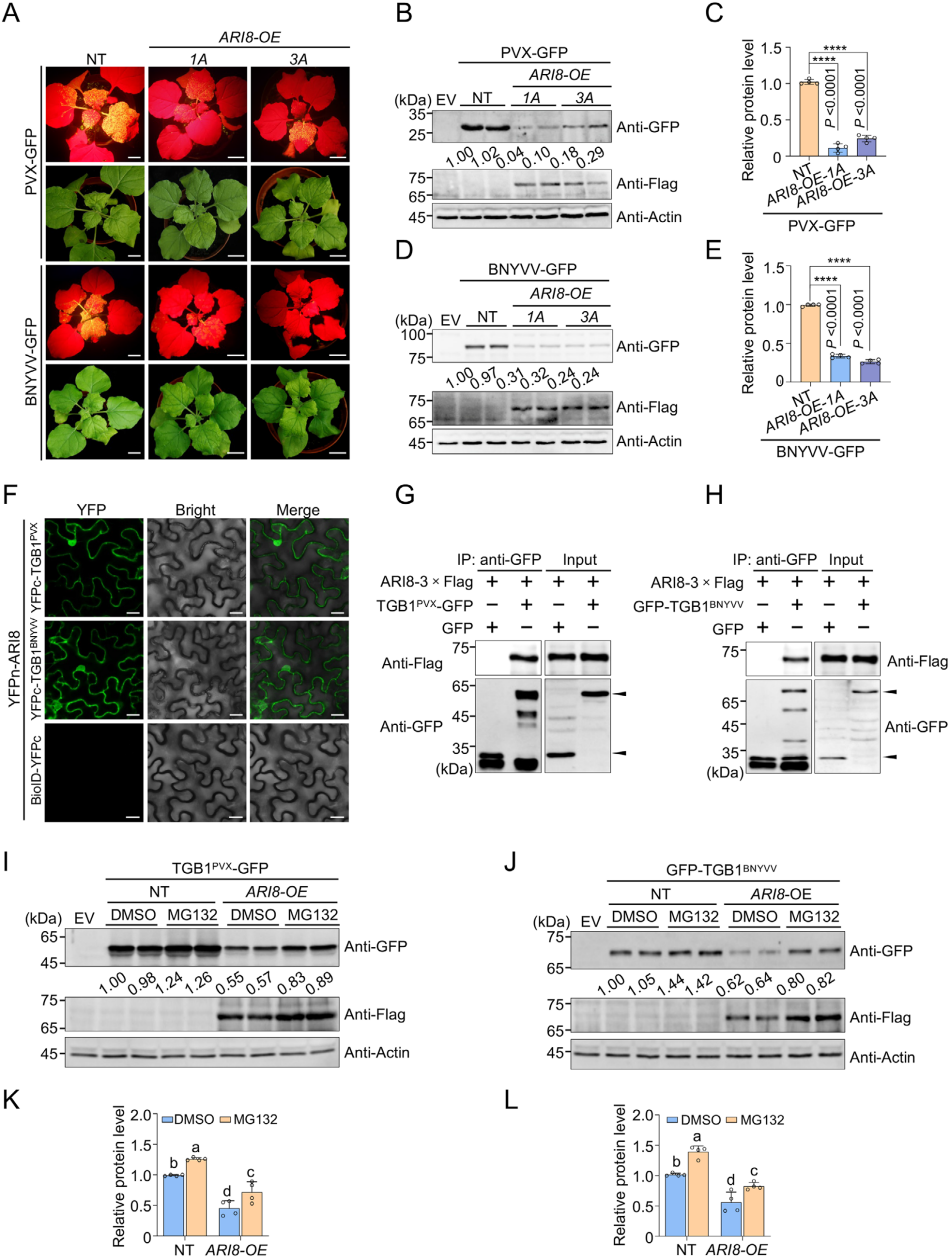
Ariadne‐like protein 8 (ARI8) plays a defensive role against other triple gene block protein (TGB)‐containing plant viruses. A) Symptoms of potato virus X (PVX) and beet necrotic yellow vein virus (BNYVV) infection in non‐transgenic (NT) and wild‐type ARI8 overexpressing (*ARI8‐OE*) *Nicotiana benthamiana* plants at 7 or 10 days post‐inoculation (dpi), respectively. Scale bar = 3.5 cm. B) Immunoblot analysis of green fluorescent protein (GFP)‐tagged PVX accumulation in the systemic leaves shown in (A). Actin was used as a loading control. C) Quantification of the GFP protein levels in (B). D) Immunoblot analysis of GFP‐tagged BNYVV accumulation in the systemic leaves shown in (A). E) Quantification of GFP protein levels in (D). F) Bimolecular fluorescence complementation (BiFC) assay showing interactions between ARI8 and TGB1 proteins derived from PVX and BNYVV (TGB1^PVX^ and TGB1^BNYVV^, respectively). ARI8 fused with the N‐terminal fragment of yellow fluorescent protein (YFPn–ARI8) was co‐expressed with TGB1^PVX^ fused with the C‐terminal fragment of YFP (YFPc–TGB1^PVX^), or YFPc–TGB1^BNYVV^ in *N. benthamiana* leaves. Reconstituted YFP fluorescence signals were visualized under confocal microscopy. Scale bars represent 20 µm. The combination of YFPn‐ARI8 and BioID–YFPc served as a negative control. G–H) Co‐immunoprecipitation (Co‐IP) analysis confirming interactions between ARI8 and TGB1^PVX^ G) or TGB1^BNYVV^ H). I, J) ARI8 mediates degradation of TGB1^PVX^ (I) or TGB1^BNYVV^ (J) protein via the 26S proteasome pathway. TGB1^PVX^–GFP or GFP–TGB1^BNYVV^ recombinant proteins were agroinfiltrated into NT or *ARI8‐OE N. benthamiana* plants. At 36 h post‐infiltration (hpi), 100 µM MG132 or dimethyl sulfoxide (DMSO) (control) was infiltrated into the same leaf regions. Approximately 12 h later, the infiltrated leaves were harvested for immunoblot analysis. Actin served as the loading control. K, L) Quantification of protein levels shown in (I) and (J), respectively. In (C), (E), and K, L), error bars represent mean ± standard deviation (*n* = 4 biological repeats). Asterisks indicate statistically significant differences based on an unpaired two‐tailed *t*‐test (****P <0.0001). Different letters above bars indicate statistically significant differences among groups as determined by Duncan's multiple range test (*P* <0.05). All experiments were independently repeated at least twice, and similar results were obtained.

To investigate whether ARI8 plays a role in regulating infections caused by other TGB‐harboring viruses, we tested its function in potato virus X (PVX) and BNYVV infections. PVX belongs to the genus *Potexviru*s of the family *Alphaflexiviridae*, and BNYVV is a member of the genus *Benyvirus* of the family *Benyviridae*. GFP‐tagged PVX (PVX–GFP) or BNYVV (BNYVV–GFP) were agroinoculated into NT and *ARI8‐OE* plants. Symptom observations revealed that, compared to NT plants, infections with both PVX–GFP and BNYVV–GFP were significantly attenuated in *ARI8‐OE* plants, as evidenced by reduced GFP fluorescence intensity in systemically infected leaves (**Figure** **6**A). Immunoblot analysis further confirmed that the accumulation of PVX (Figure 6B,C) and BNYVV (Figure 6D,E) was significantly reduced in the systemic leaves of *ARI8‐OE* plants compared with that in NT plants. These results indicate that ARI8 negatively regulates infections caused by other TGB1‐encoding viruses.

To determine whether ARI8 interacts with the TGB1 proteins encoded by BNYVV and PVX, we performed BiFC and Co‐IP assays, which consistently demonstrated that ARI8 interacts with the TGB1 proteins of both PVX (Figure 6F,G) and BNYVV (Figure 6F,H). Immunoblot analysis confirmed the protein expression in the BiFC assay (Figure S5C, Supporting Information). In WT and *ARI8‐OE* transgenic plants, TGB1^PVX^–GFP and GFP–TGB1^BNYVV^ were transiently expressed via agroinfiltration, followed by the additional infiltration of the 26S proteasome inhibitor MG132 into pre‐infiltrated leaves 12 h before sampling. Immunoblot analysis revealed that the MG132 treatment increased the accumulation of both PVX‐ (Figure 6I,K) and BNYVV‐encoded TGB1 proteins in WT plants (Figure 6J,L) compared with that in plants subjected to the DMSO control treatment, suggesting that these TGB1 proteins may undergo ubiquitination. Moreover, the accumulation levels of TGB1 proteins from PVX and BNYVV were significantly reduced in *ARI8‐OE* plants compared to those in WT plants, whereas MG132 treatment partially restored their levels compared to those in the DMSO controls (Figure 6I–L). These results suggest that ARI8 targets other viral TGB1 proteins, in addition to the BSMV‐encoded TGB1, for degradation, thereby suppressing viral infections.

## 3 Discussion

UPS, a key pathway for maintaining cellular physiological balance, plays a vital role in plant growth, development, and responses to various environmental stresses.^[^ ^36^ ^]^ Over the past 30 years, the involvement of UPS in plant–microbe interactions, particularly with viruses, has been extensively studied. UPS functions as a double‐edged sword in viral pathogenesis. Conversely, UPS functions as an essential component of the plant antiviral defense system by targeting virus‐encoded proteins for ubiquitination and subsequent degradation.^[^ ^9^, ^10^, ^37^, ^38^ ^]^ In contrast, viruses manipulate the UPS machinery to promote or inhibit the degradation of specific host proteins, creating a favorable intracellular environment for viral infection.^[^ ^39^, ^40^, ^41^ ^]^ Various plant virus‐encoded proteins, including CPs,^[^ ^42^ ^]^ MPs,^[^ ^12^, ^13^ ^]^ RNA‐dependent RNA polymerases,^[^ ^43^, ^44^ ^]^ and viral suppressors of RNA silencing,^[^ ^33^, ^45^, ^46^ ^]^ have been reported to undergo E3 ligase‐mediated ubiquitination and proteasomal degradation. However, only a few E3 ligases have been identified that directly ubiquitinate viral proteins, likely because of the weak and transient interactions between E3 ligases and their substrates, as well as the rapid turnover of target proteins, which together make it challenging to capture a comprehensive interactome during UPS–virus interactions. Here, we identified an RBR‐type E3 ligase, ARI8, which directly interacts with the TGB1 MP of BSMV and mediates its degradation via the 26S proteasome pathway, thereby impairing viral movement and suppressing BSMV infection. These findings advance our understanding of the critical role of UPS in plant antiviral defenses.

Plant viral MPs are primarily classified into two types: a single cell‐to‐cell MP of the “30K” superfamily and those encoded by a TGB.^[^ ^47^, ^48^, ^49^ ^]^ Ubiquitination has been reported for MPs of both types.^[^ ^11^, ^14^ ^]^ As early as 1990, Lehto et al. conducted a time‐course analysis of TMV MP accumulation in intact tobacco leaves, revealing that MP levels initially increase but later decline as TMV infection progresses. In contrast, other TMV‐encoded proteins, such as CP and 126 K protein, showed continuous accumulation until reaching a plateau.^[^ ^50^ ^]^ The mechanism underlying this unique accumulation pattern of MP remains poorly understood. Interestingly, although the BSMV TGB1 protein belongs to a different class than the TMV 30 K MP, we observed a similar accumulation pattern during BSMV infection: TGB1 levels initially increased and then declined. In contrast, other BSMV‐encoded proteins, including γb and CP, continued to accumulate, and their accumulation levels plateaued over time (Figure 1A,B). This striking similarity to the TMV infection pattern suggests the existence of a conserved regulatory mechanism that modulates MP levels during viral infection.

Reichel & Beachy demonstrated that the 26S proteasome degrades TMV 30 K MP from mobile complexes associated with the endoplasmic reticulum (ER).^[^ ^11^ ^]^ Similarly, PVX TGBp3, which is also associated with the ER network, undergoes degradation through the 26S proteasome, and its turnover is impacted by S‐phase kinase‐associated protein 1 (SKP1), a component of the SKP1–cullin1–F‐box complex.^[^ ^51^, ^52^, ^53^ ^]^ Recently, Guo et al. reported that the ER‐associated degradation (ERAD)‐localized E3 ligase 3‐hydroxy‐3‐methylglutaryl coenzyme A reductase degradation 1 (HRD1) targets and mediates the ubiquitination of TGB MPs from both BNYVV and PVX.^[^ ^14^ ^]^ As infections caused by TMV, BNYVV, and PVX induce ER stress,^[^ ^53^, ^54^, ^55^ ^]^ these findings highlight the essential role of the ERAD system in antiviral defense by degrading viral MPs.

In the present study, we identified a nucleocytoplasmically localized RBR‐type E3 ligase, ARI8, which is recruited to the PD upon BSMV infection and mediates the ubiquitination and degradation of the TGB1 protein (Figures 2A and 4; Figure S4, Supporting Information). Our findings revealed an antiviral mechanism in which the UPS pathway, which is distinct from the ERAD system, suppresses viral infection by specifically targeting viral MPs. Considering that the movement of BSMV is also associated with the ER/actin network,^[^ ^34^ ^]^ we cannot fully exclude the possibility that BSMV‐encoded TGB proteins may also act as substrates for ERAD‐related E3 ligases, such as HRD1. It is reasonable to propose that targeting the same viral protein using multiple E3 ligases enables plants to exert more precise spatial and temporal control over viral infections, reflecting the long‐term evolutionary arms race between plants and viruses. Since BSMV TGB proteins facilitate cell‐to‐cell movement through the PD, the recruitment of ARI8 to the PD to mediate TGB1 degradation may help protect host cellular integrity, ensure cell survival, or serve as a defense mechanism to restrict the spread of BSMV to neighboring cells. The changes in the subcellular localization of ARI8 post‐BSMV infection (Figure 2A; Figure S4, Supporting Information) and its self‐ubiquitinating ability (Figures 4L and 5D) suggest that BSMV infection likely triggers dynamic changes in ARI8 at protein and cellular levels, enabling it to negatively regulate BSMV infection via the targeted degradation of TGB1.

RBR‐type E3 ligases are highly conserved in yeasts, humans, and plants (Figures S1 and S2, Supporting Information). In mice, the RBR‐type E3 ligase ARIH1 interacts with cyclic GMP‐AMP synthase (cGAS) to catalyze mono‐ISGylation, promoting cGAS oligomerization, thereby enhancing antiviral immunity against herpes simplex virus 1.^[^ ^56^ ^]^ Although two recent studies have reported that plant RBR‐type E3 ligases can target endogenous proteins, such as the jasmonate signaling repressor NINJA3^[^ ^27^ ^]^ and the NRC4 protein,^[^ ^28^ ^]^ it remains unknown whether RBR‐type E3 ligases can directly target and degrade pathogen‐encoded proteins. Our study addresses this gap by demonstrating that ARI8 directly targets the viral MP, TGB1, for ubiquitination and subsequent degradation via the 26S proteasome, thereby inhibiting viral movement and systemic infection. Furthermore, ARI8 confers resistance not only to BSMV but also to BNYVV and PVX (Figure 6A–E), suggesting a general role for ARI8 in defending against viruses encoding a TGB1 protein. Whether ARI8 also functions against viruses that do not encode TGB1 remains an open question worthy of further investigation. Taken together, our findings, along with previous studies,^[^ ^27^, ^28^ ^]^ indicate that RBR‐type E3 ligases can regulate plant defense either by modulating host defense signaling pathways or by directly targeting pathogen‐encoded proteins, highlighting the functional diversity of RBR‐type E3 ligases in plant‐pathogen interactions. Additionally, we observed that *ARI8‐OE* plants exhibited a dwarf phenotype, which may be related to the immune activation triggered by ARI8 overexpression or the degradation of proteins involved in plant development.

In summary, herein, we revealed, for the first time, the functional role of the RBR‐type E3 ligase ARI8 in plants, providing a potential molecular target for the development of antiviral strategies. In addition to TGB1, the full spectrum of ARI8 substrates *in planta* remains unknown and warrants further investigation. Additionally, our previous studies have demonstrated that BSMV TGB1 undergoes phosphorylation.^[^ ^57^ ^]^ Whether there is a crosstalk between the phosphorylation and ubiquitination of BSMV‐encoded TGB1 is another intriguing question that deserves future exploration.

## 4 Experimental Section

### Plant Growth Conditions

*Nicotiana benthamiana* plants were grown in a growth chamber maintained at 23–25°C under a 16 h light:8 h darkness photoperiod.

### Plasmid Constructions

In this study, unless otherwise specified, DNA fragments were inserted into vectors using the Seamless Assembly Cloning Kit (Catalog number C5891‐50; Clone Smarter Technologies Inc., Houston, USA). The plasmids for BSMV, BSMV‐based BiFC system, BSMV_3×Flag–TGB1_, BSMV_γb–3×Flag_, and dfBSMV have been described in this previous studies.^[^ ^33^, ^34^ ^]^ For transient expression in *N*. *benthamiana*, full‐length DNA fragments encoding *ARI8* and *Hv* *ARI8* were amplified and inserted into the *Sal*I‐ and *Bam*HI‐digested pGD‐6×Myc vector, a modified version of the pGD vector.^[^ ^58^ ^]^ DNA fragments encoding *ARI8*, *ARI8^C311S^*, *GFP*, and *TGB1* were amplified and cloned into *Kpn*I‐ and *Spe*I‐digested pMDC32‐3×FLAG vectors.^[^ ^59^ ^]^ For Co‐IP assays, DNA fragments encoding *ARI8*, *TGB1*, or *TGB1^BNYVV^* were amplified and cloned into the *Hind*III‐digested pGDG vector.^[^ ^58^ ^]^ DNA fragments encoding *TGB1* ^*PVX*^ were amplified and cloned into the *Sal*I‐digested pGDGm vector, a modified version of pGDG^[^ ^58^ ^]^ containing a three‐glycine linker and a C‐terminal GFP fusion.

For subcellular localization, *ARI8* was cloned into the *Sal*I‐digested pGD3G‐mCherry vector, a modified version of the pGD vector^[^ ^58^ ^]^ containing a three‐glycine linker and a C‐terminal mCherry fusion. For BiFC assays, the DNA fragments encoding *ARI8* and *HvARI8* were inserted into the pSPYNE(R)173 vector, and DNA fragments encoding *TGB1* ^*BSMV*^, *TGB1* ^*BNYVV*^, and *TGB1* ^*PVX*^ were inserted into the pSPYCE(MR) vector^[^ ^60^ ^]^ to generate YFPc‐fused TGB1. For split‐luciferase assays, DNA fragments encoding *GUS*, *ARI8*, *ARI8^C311S^*, and *TGB1* were amplified and inserted into *Bam*HI‐ and *Sal*I‐digested pCAMBIA1300‐nLuc or pCAMBIA1300‐cLuc vectors,^[^ ^61^ ^]^ respectively, to generate the cLuc–TGB1, TGB1–nLuc, GUS–nLuc, ARI8–nLuc, cLuc‐ARI8 and cLuc–ARI8^C311S^ constructs.

For the GST pull‐down assays, the DNA fragments encoding *TGB1* were amplified and inserted into the *Bam*HI‐ and *Sal*I‐digested pGEX‐KG vectors to express the GST‐tagged TGB1 protein in *E. coli*. The *ARI8* DNA fragment was cloned into *Kpn*I‐ and *Xho*I‐digested pET30a (+) plasmids for the expression of His‐tagged ARI8. GFP–His protein expression was measured as described previously.^[^ ^62^ ^]^ For CRISPR‐mediated gene editing, a single guide RNA (sgRNA) targeting the loci of both *ARI8* paralogs, Niben101Scf03512g02004.1, and Niben101Scf08947g02007.1, was designed using online CRISPR RGEN Tools (http://www.rgenome.net/cas‐designer/). The sgRNA sequences were cloned into the pKSE401 vector^[^ ^63^ ^]^ to generate the pKSE401‐ARI8 construct.

The primer sequences used for plasmid construction were listed in Table S2 (Supporting Information). All constructs were verified by DNA sequencing to ensure the accuracy of the plasmids.

### Generation of Transgenic Plants

Transgenic *N. benthamiana* plants were generated using the *Agrobacterium*‐mediated leaf disk transformation method. The construct was introduced into the *Agrobacterium tumefaciens* strain EHA105 using the freeze‐thaw method. Specifically, *ARI8‐OE* and *ARI8^C311S^‐OE* lines in the wild‐type (WT) *N*. *benthamiana* background were prepared using *Agrobacterium* carrying pMDC32‐ARI8–3×Flag and pMDC32‐ARI8^C311S^–3×Flag, respectively. *ARI8‐KO* lines in the *sfGFP1*–*10^OPT^*‐transgenic *N*. *benthamiana* (*G10*) background were generated using *Agrobacterium* containing pKSE401‐ARI8. The *sfGFP1*–*10^OPT^* transgenic *N. benthamiana* (*G10*) plants have been described earlier.^[^ ^33^ ^]^

Healthy leaves were harvested from 4‐to 6‐week‐old donor plants and surface‐sterilized. Leaf disks (≈0.5–1 cm in diameter) were excised and immersed in an *Agrobacterium tumefaciens* suspension harboring the desired construct, prepared at an optical density at 600 nm (OD_600_) of 0.5–1.0 in infiltration medium containing MgCl_2_ (10 mmol L^−1^), MES (10 mmol L^−1^, pH 5.6), and acetosyringone (100 µmol L^−1^). The disks were then incubated with *Agrobacterium* for 10–15 min with gentle agitation. After co‐cultivation on solid Murashige and Skoog medium supplemented with appropriate hormones and without antibiotics for 2 days in the dark at 22–25°C, the leaf disks were transferred to selective regeneration medium containing antibiotics to eliminate *Agrobacterium* and select for transformed cells. The disks were maintained under controlled light conditions (16 h light:8 h dark) to induce shoot regeneration. The regenerated shoots were rooted and transferred to soil under controlled growth conditions. Positive transgenic lines of *ARI8‐OE* and *ARI8^C311S^‐OE N*. *benthamiana* were screened by immunoblot analysis, whereas gene editing in *ARI8‐KO* lines was confirmed by genomic DNA extraction, followed by DNA sequencing at the *ARI8* loci. Homozygotes with frameshift mutations in both *ARI8* paralogs were selected as *ARI8‐KO* lines for subsequent experiments.

### Viral Inoculation

For BSMV inoculation, *Agrobacterium* mixtures containing plasmids pCB301‐α, pCB301‐β, and pCB301‐γ^[^ ^33^ ^]^ or their derivatives were mixed at equal concentration and infiltrated into 4‐ to 6‐week‐old *N. benthamiana* plants as described previously.^[^ ^64^ ^]^

### BiFC Assays and Subcellular Localization Analysis

*Agrobacterium* harboring various BiFC constructs or plasmids expressing different fluorescent protein fusions were mixed and co‐infiltrated into the leaves of 4‐week‐old *N*. *benthamiana*. At 48 h post‐infiltration (hpi), the leaf tissues were observed using a Carl Zeiss LSM880 confocal microscope according to previously described procedures.^[^ ^65^ ^]^ GFP and YFP fluorescence signals were excited at 488 nm, and mCherry and aniline blue staining were visualized at 561 and 405 nm, respectively. The working concentration of the aniline blue used in this study was 0.025 mg mL^−1^.^[^ ^66^ ^]^

### Firefly LCI Assays

LCI assays were performed as previously described.^[^ ^61^, ^67^ ^]^ *Agrobacterium* mixtures harboring nLuc and cLuc fusion constructs were co‐infiltrated into *N. benthamiana* leaves. At 48 hpi, leaf samples were collected, sprayed with luciferin (1 mmol L^−1^), and luminescence was captured using the Night SHADEs LB985 in vivo Plant Imaging System (Berthold, Germany). The fluorescence intensity was quantified using ImageJ software.

### Co‐IP Assays

Co‐IP assays were performed as previously described, with minor modifications.^[^ ^68^ ^]^ *Agrobacterium* mixtures harboring the indicated constructs were co‐infiltrated into *N. benthamiana* leaves. At 48 hpi, 3–4 g of leaves was harvested, flash‐frozen in liquid nitrogen, and ground into a fine powder. Total protein was extracted using ice‐cold lysis buffer containing Tris‐HCl (25 mmol L^−1^, pH 7.5), NaCl (150 mmol L^−1^), EDTA (1 mmol L^−1^), and glycerol (10%). The homogenate was incubated on ice for 45 min, followed by centrifugation at 8000 ×*g* for 15 min at 4°C to remove the insoluble debris. The resulting supernatant was incubated with anti‐Flag or anti‐GFP antibody‐conjugated agarose beads at 4°C for 3–4 h with gentle agitation. After incubation, the beads were collected by centrifugation at 800 ×*g* for 1 min at 4°C and washed extensively with IP buffer containing Tris‐HCl (25 mmol L^−1^, pH 7.5), NaCl (150 mmol L^−1^), EDTA (1.0 mmol L^−1^), glycerol (10%), and NP‐40 (0.1%, v/v) to remove non‐specific binding. The immunoprecipitated products were separated by sodium dodecyl sulfate–polyacrylamide gel electrophoresis (SDS–PAGE) and analyzed by immunoblotting with anti‐FLAG (1:5000 dilution; Catalog number A2220; Sigma‐Aldrich, St. Louis, MO, USA) or anti‐GFP (1:5000 dilution; Catalog number BE2002; Bioeasytech, Beijing, China) antibodies.

### GST Pull‐Down Assays

GST pull‐down assays were conducted as previously described with minor modifications.^[^ ^69^ ^]^ Purified 6 µg of GST‐tagged TGB1 were incubated with 5 µg of ARI8 or His‐tagged GFP (negative control) in binding buffer containing Tris‐HCl (pH 7.5‐8.0, 50 mmol L^−1^), NaCl (200 mmol L^−1^), DTT (5.0 mmol L^−1^), glycerol (0.2%, v/v), Triton X‐100 (0.6%, v/v), and 1×proteinase inhibitor cocktail. The mixture was incubated with 30 µL of GST agarose beads for 3 h at 4°C with gentle agitation. The beads were collected by centrifugation at 800 ×*g* for a minute, and the supernatant was discarded. The beads were washed six times with wash buffer containing Tris‐HCl (50 mmol L^−1^, pH 7.5), NaCl (250 mmol L^−1^), glycerol (0.2%, v/v), and Triton X‐100 (0.6%, v/v) to remove non‐specifically bound proteins. The pull‐down products were analyzed by immunoblotting with anti‐GST (1:5000; Catalog number A00866; Genscript Biotech Corporation, Nanjing, China) or anti‐His (1:5000; Catalog number BE7001; EASYBIO, Beijing, China) antibodies.

### Cell‐Free Protein Degradation Assays

The Cell‐free protein degradation assays were performed as previously described with minor modifications.^[^ ^33^ ^]^ Total proteins were extracted using a non‐denaturing buffer containing Tris‐HCl (50 mmol L^−1^, pH 8.0), EDTA (10 mmol L^−1^, pH 8.0), sucrose (0.5 mol L^−1^), MgCl_2_ (1.0 mmol L^−1^), and dithiothreitol (DTT, 5.0 mmol L^−1^), with the proteasome inhibitor MG132 (50 µmol L^−1^) or DMSO (control). To monitor protein degradation, the extracts were supplemented with 0.5 mmol L^−1^ CHX to inhibit *de novo* protein synthesis and ATP (10 mmol L^−1^) to facilitate proteasomal activity. Reaction mixtures were incubated at 25°C for different time periods, and the levels of TGB1‐3×Flag were analyzed by immunoblotting using an anti‐TGB1 antibody.

Alternatively, total proteins from *G10* or *ARI8‐KO N. benthamiana* leaves were extracted using native buffer as described above, and purified GST‐TGB1 protein from *E. coli* was added to the leaf extracts. The reaction mixtures were incubated at 25°C for different periods, and TGB1 protein levels were assessed by immunoblotting using an anti‐TGB1 antibody.

### In Vivo Ubiquitination Assay

In vivo ubiquitination assays were performed as previously reported.^[^ ^33^ ^]^ TGB1–3×Flag, BSMV_3×Flag–TGB1_, or GFP–TGB1 was co‐expressed with HA‐tagged *At*UBQ11 or Flag‐tagged *At*UBQ11 in *N. benthamiana* leaves via agroinfiltration. Leaf tissues were harvested on different days post‐infiltration, flash‐frozen in liquid nitrogen, and ground into a fine powder. Total proteins were extracted using a buffer containing Tris‐HCl (25 mmol L^−1^, pH 7.5), NaCl (150 mmol L^−1^), EDTA (1.0 mmol L^−1^), glycerol (10%, v/v), DTT (10 mmol L^−1^), NP40 (0.1%, v/v), PVP40 (2%, m/v), 1 × proteinase inhibitor cocktail, N‐ethylmaleimide (NEM, 10 mmol L^−1^; Catalog number E1271; Sigma‐Aldrich), ubiquitin aldehyde (10 mmol L^−1^; Catalog number 662 056; EMD Millipore Corporation), PMSF (1.0 mmol L^−1^) and MG132 (50 µmol L^−1^). For immunoprecipitation, the extracts were incubated with anti‐Flag (Catalog number GNI4510‐FG; GNI; or Catalog number KTSM1308; AlpaLifeBio) or anti‐GFP agarose beads at 4°C for 3 h. Beads were sequentially washed under cold conditions three times with W1 buffer containing Tris‐HCl (25 mmol L^−1^, pH 7.5), NaCl (150 mmol L^−1^), EDTA (1.0 mmol L^−1^), glycerol (10%, v/v), NP40 (0.1%, v/v), 1× proteinase inhibitor cocktail, NEM (10 mmol L^−1^; Catalog number E1271; Sigma), ubiquitin aldehyde (10 mmol L^−1^; Catalog number 662 056; EMD Millipore Corporation), PMSF (1.0 mmol L^−1^) and MG132 (50 µmol L^−1^), and thrice with W2 buffer containing Tris‐HCl (25 mmol L^−1^, pH 7.5), NaCl (150 mmol L^−1^), EDTA (1.0 mmol L^−1^), glycerol (10%, v/v), NP40 (0.1%, v/v), NEM (10 mmol L^−1^; Catalog number E1271; Sigma), ubiquitin aldehyde (10 mmol L^−1^; Catalog number 662 056; EMD Millipore Corporation) and PMSF (1.0 mmol L^−1^). Ubiquitin‐conjugated proteins were separated using by SDS–PAGE and detected by immunoblotting with an anti‐*At*UBQ11 antibody.

### In Vitro Ubiquitination Assay

An in vitro ubiquitination assay was performed using a bacterial reconstitution system, as described previously.^[^ ^35^ ^]^ Plasmids, including pACYCDuet‐ARI8–Myc, pACYCDuet‐ARI8–Myc–*At*UBC8‐S, pMAL‐MBP–TGB1–HA, and pCDFDuet‐MBP–TGB1–HA–*At*UBA1‐S, together with pCDFDuet‐*At*UBA1–S, pACYCDuet‐*At*UBC8–S, and pET28a‐Flag–UBQ, were transformed into *E. coli* BL21 (DE3). These bacteria containing different combinations of expression vectors were cultured at 37°C until the OD_600_ reached 0.4–0.6, followed by the addition of isopropyl β‐D‐1‐thiogalactopyranoside (0.5 mmol L^−1^). The culture was then transferred to a shaker at 28°C for 10–12 h. Finally, the culture was incubated at 4°C overnight. Protein extracts were separated by SDS–PAGE and subjected to immunoblot analysis using anti‐HA (Catalog number C29F4; Cell Signaling, Danvers, Massachusetts, USA), anti‐Flag (1:5000; Catalog number A2220; Sigma‐Aldrich, St. Louis, MO, USA), or anti‐Myc (Catalog number M047‐3; Medical & Biological Laboratories, Tokyo, Japan) antibodies.

### Sequence Alignment and Phylogenetic Analyses

*NbARI8* orthologs from *Nicotiana tabacum*, *Arabidopsis thaliana*, *H. vulgare*, *Brachypodium distachyon*, *Triticum aestivum*, *Oryza sativa*, *Zea mays L*., *Solanum lycopersicum L*., *Solanum tuberosum*, *Setaria italica*, and *Glycine max (L.) Merr*. were identified using the Basic Local Alignment Search Tool (BLAST) against the National Center for Biotechnology Information, Ensembl Plants (http://plants.ensembl.org/index.html), and the *N. benthamiana* Sol Genomics Network database (https://solgenomics.net/organism/Nicotiana_benthamiana/genome). Sequence alignment was performed using DNAMAN software (version 6.0), and the phylogenetic analysis of putative *ARI8* gene sequences from various plant species was performed using MEGA11 software.^[^ ^70^ ^]^

### Statistical Analyses

Protein abundance from immunoblots was quantified by measuring band intensities with ImageJ software (NIH, Bethesda, Maryland, USA) and normalized to loading controls, typically using data from 3–4 independent biological replicates. To assess the movement of BSMV or its derivatives, red fluorescence was used to mark the initial inoculation area, whereas green fluorescence indicated the regions of BSMV infection. The area exhibiting green fluorescence beyond the red fluorescence represented the movement zone of the virus. The size of the GFP‐only region was quantified and normalized by dividing the area of this region by the area of the corresponding red fluorescent region. Experimental data were statistically analyzed using the Student's *t*‐test (n.s. = not significant, *P* >0.05, *P <0.05, **P <0.01, ***P <0.001, and ****P <0.0001) or Dunnett's multiple comparison test (different letters indicate significant differences, *P* <0.05) using GraphPad Prism 9 software.

## Conflict of Interest

The authors declare no conflict of interest.

## Author Contributions

Y.Z. and W.L. conceived the study. W.L., C‐C.Z., J.D., C‐Y.Z., Z.L., and D.Z. performed the experiments. W.L., C‐C.Z., X.Z., Y.Z., and Z.W. analyzed the data. Y.Z., X‐F.Z., M.Y., D‐S.L., and D.L. supervised the project. W.L. and Y.Z. wrote the manuscript. All authors have proofread and approved the manuscript.

## Supporting information



Supporting Information

Supplementary TableS1

## Data Availability

The data that support the findings of this study are available in the supplementary material of this article.
