# Supplementary material for: RING‐Between‐RING‐Type E3 Ligase Ariadne‐Like Protein 8 Negatively Regulates Plant Virus Infection by Targeting a Viral Movement Protein
Source: Adv Sci (Weinh). 2025 Sep 4;12(44):e09942. doi: 10.1002/advs.202509942 (PMC12667515; doi:10.1002/advs.202509942)
Supplement: Supplementary file 1 — Supporting Information [file ADVS-12-e09942-s001.docx]

**Supporting Information**

**RING-between-RING-Type E3 Ligase Ariadne-Like Protein 8 Negatively Regulates Plant Virus Infection by Targeting a Viral Movement Protein**

Wenli Li^1^, Chenchen Zhong^1^, Jiangning Duan^1^, Changyi Zhan^1^, Zhaolei Li^1^, Xinyu Zhang^1^, Dingliang Zhang^1^, Deshui Liu^2^, Zhiyan Wen^3^, Xiaofei Zhao^1^, Meng Yang^1^, Dawei Li^1^, Yongliang Zhang*

^1^ State Key Laboratory of Plant Environmental Resilience, College of Biological Sciences, China Agricultural University, Beijing 100193, China

^2^ Beijing Life Science Academy, Beijing 102200, China

^3^ HouJi Laboratory in Shanxi Province, College of Agricultural, Shanxi Agricultural University, Taiyuan, China

* To whom correspondence should be addressed.

**Email:** cauzhangyl@cau.edu.cn

**Table of content**

Figure S1………………………………………………………………………………3

Figure S2………………………………………………………………………………4

Figure S3………………………………………………………………………………5

Figure S4………………………………………………………………………………6

Figure S5………………………………………………………………………………7

Figure S6………………………………………………………………………………8

Figure S7………………………………………………………………………………9

Figure S8……………………………………………………………………………...10

Figure S9……………………………………………………………………………...11

Table S1………………………………………………………………………………12

Table S2………………………………………………………………………………12

**
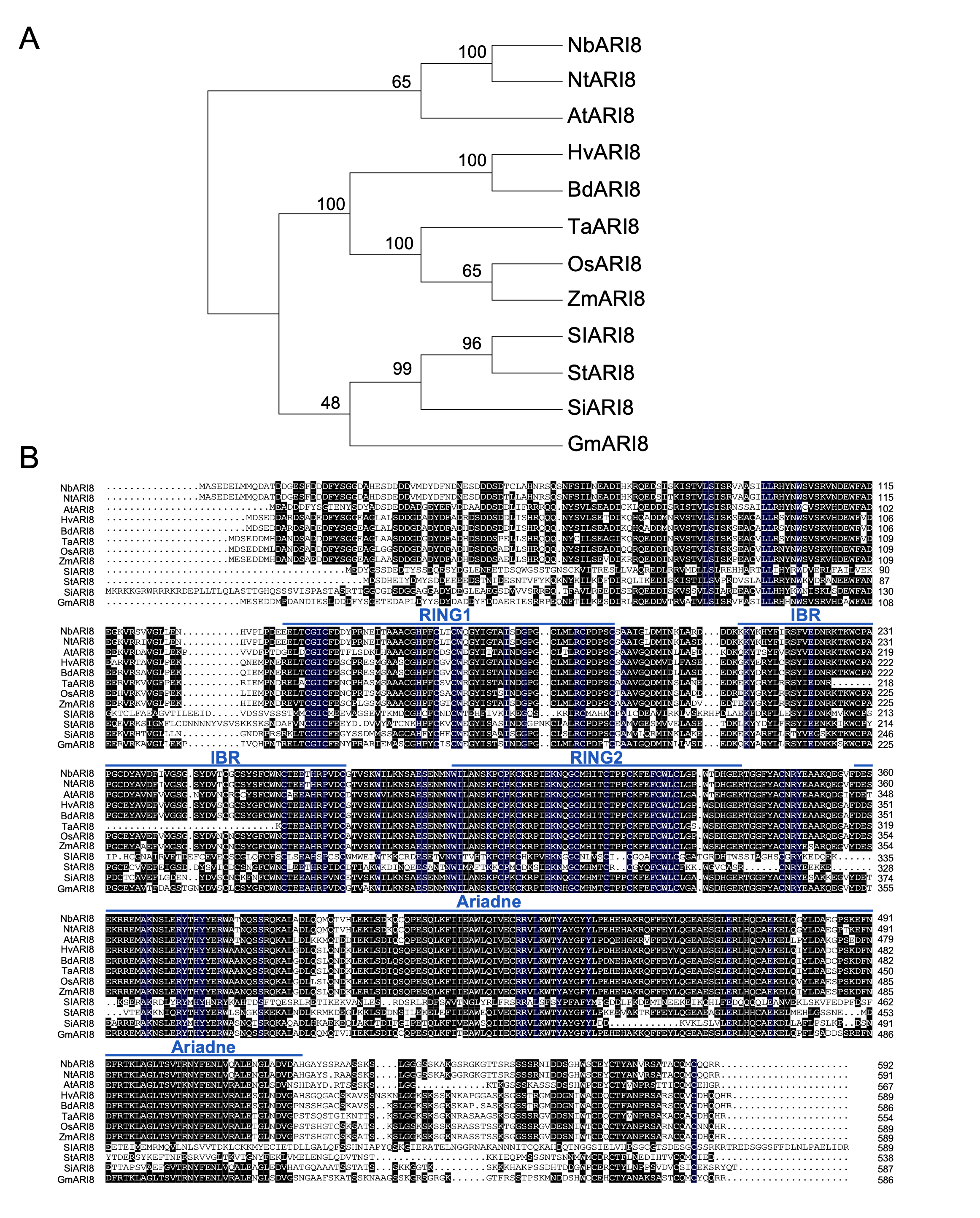
**

**Figure S1.** Phylogenetic analysis and sequence alignment of putative Ariadne-like protein 8 (ARI8) orthologs in monocot and dicot plants. A) Phylogenetic tree of ARI8 orthologs from selected monocot and dicot species. B) Multiple sequence alignment of the ARI8 orthologs used in (A).


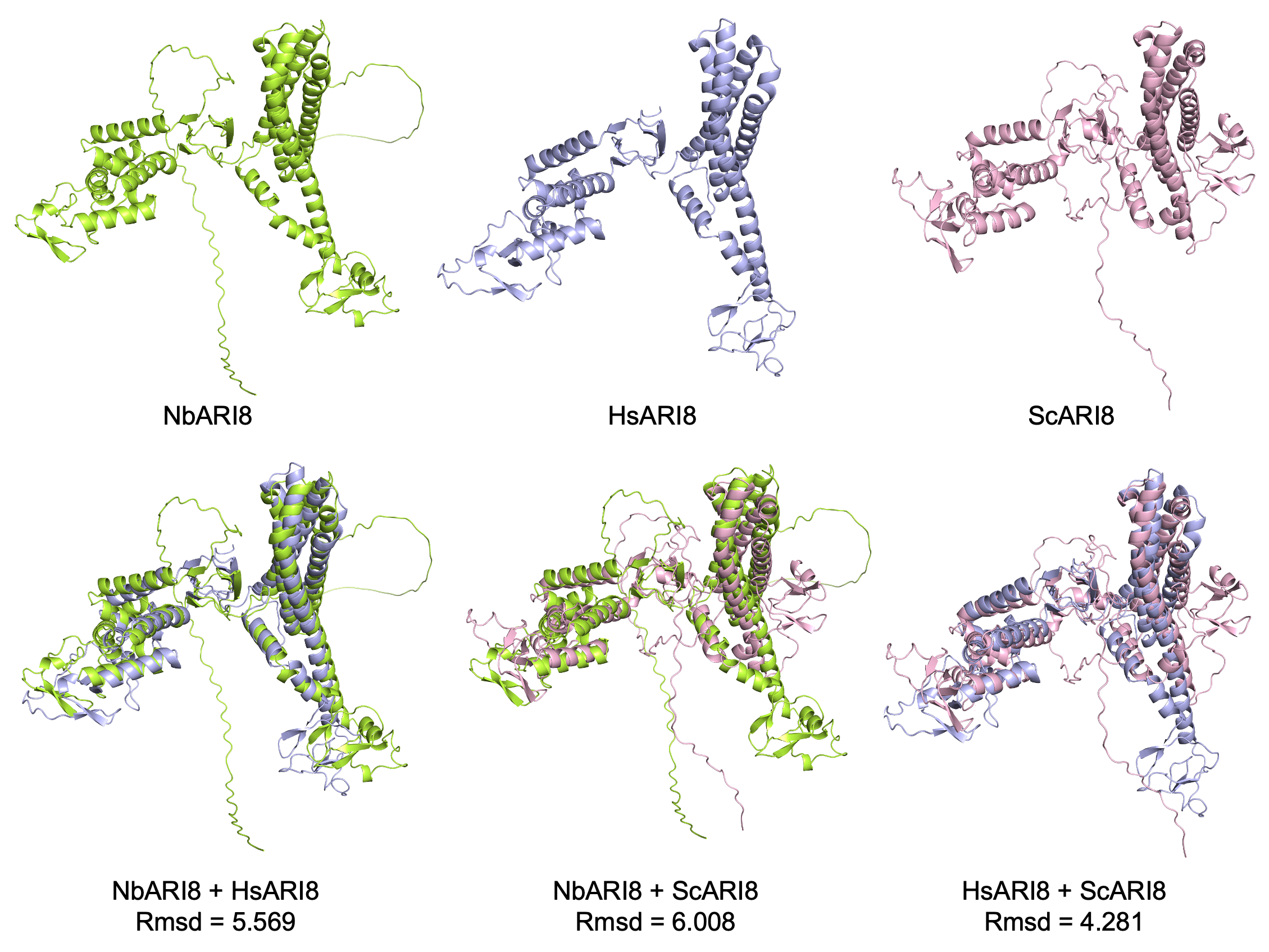


**Figure S2.** Structural comparison of Ariadne-like protein 8 (ARI8) orthologs from different organisms as predicted by AlphaFold 3. Predicted models of *Nicotiana benthamiana* ARI8 (*Nb*ARI8) are shown in green, human *Hs*ARI8 in light purple, and yeast *Sc*ARI8 in pink. The root-mean-square deviation values indicate the degree of structural similarity between pairs of models, with lower values reflecting a closer match.


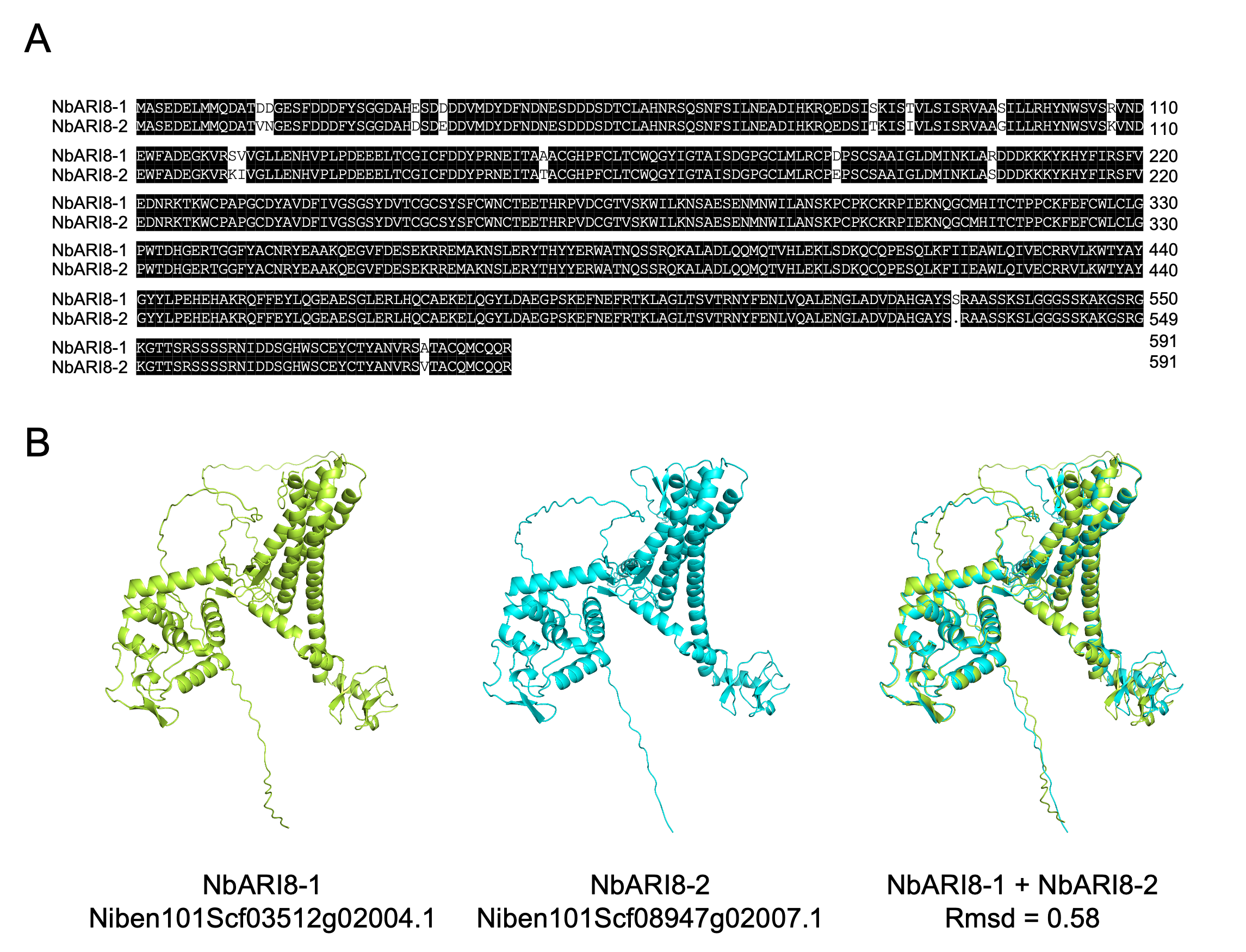
 **Figure S3.** Sequence alignment and structural comparison of putative Ariadne-like protein 8 (*ARI8*) paralogs in *Nicotiana benthamiana*. A) Amino acid sequence alignment of two *ARI8* paralogs from *N. benthamiana*. B) Structural comparison of *ARI8* paralogs from *N. benthamiana*, as predicted by AlphaFold 3.

**
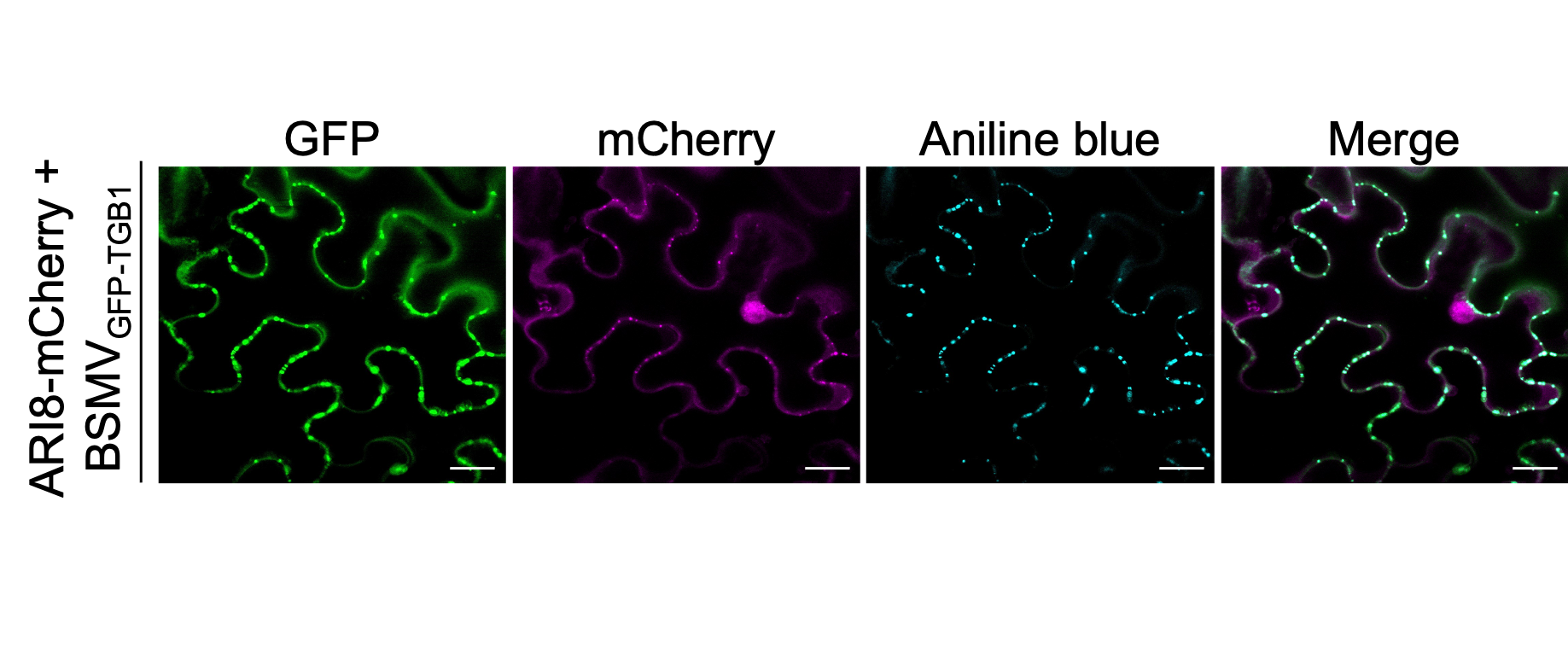
**

**Figure S4.** Fluorescence microscopy shows the co-localization of Ariadne-like protein 8 (ARI8) and triple gene block 1 (TGB1) proteins at the plasmodesmata in *Nicotiana benthamiana* during barley stripe mosaic virus (BSMV) infection. Scale bars represent 20 µm.

**
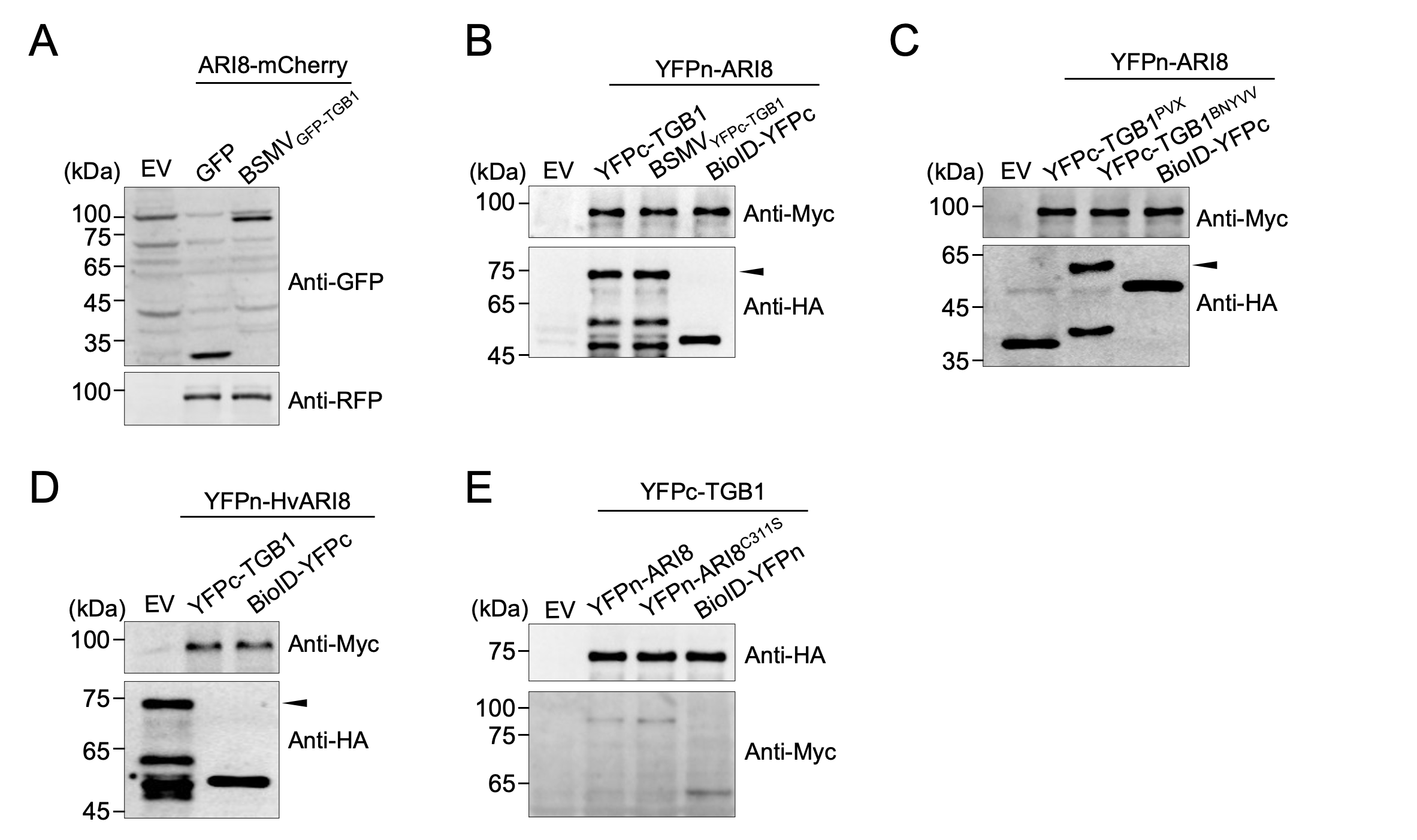
**

**Figure S5.** Immunoblot analysis to validate protein expression shown in Figures 2A, 2D, 6F, S8A, and S9B.

**
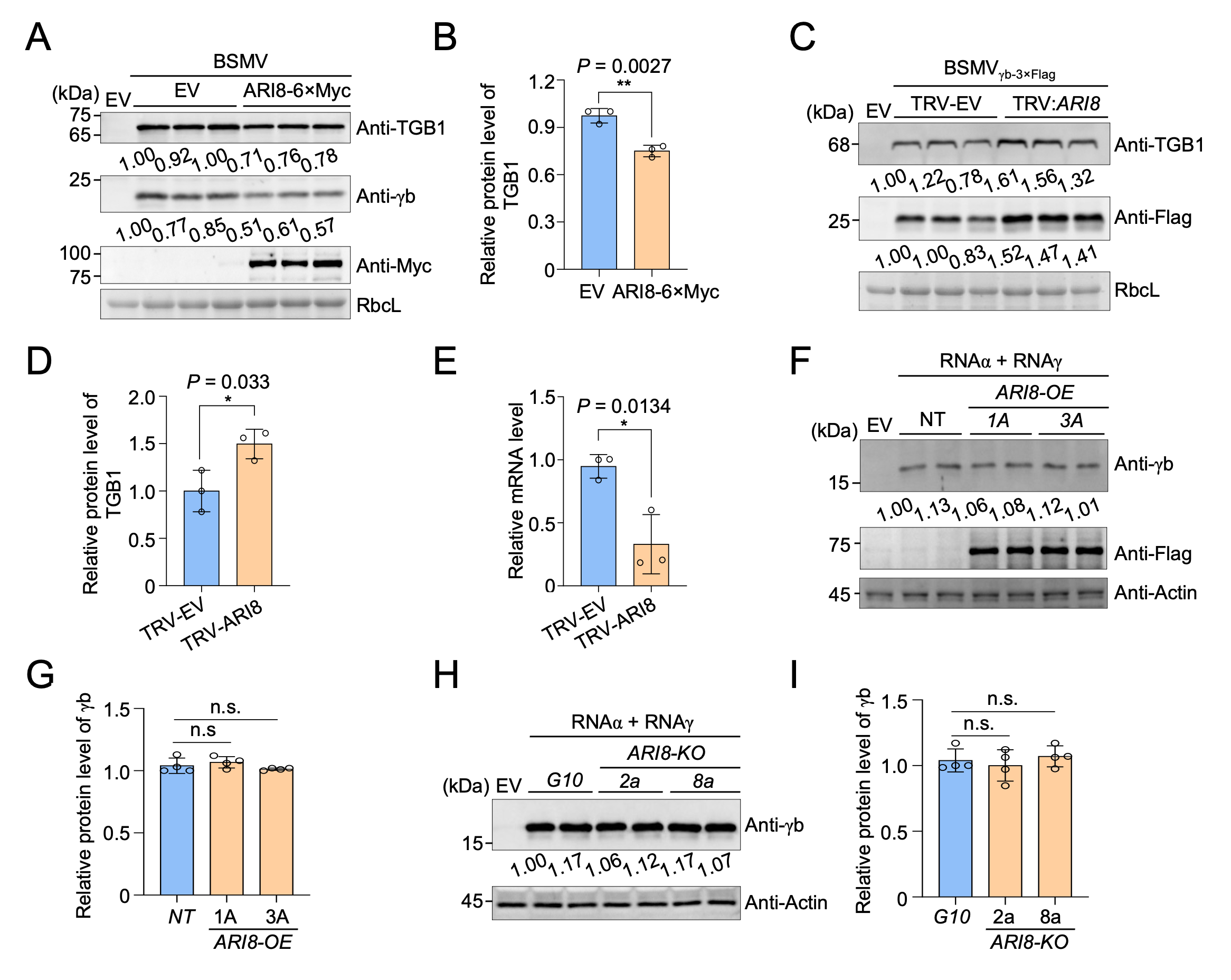
**

**Figure S6.** Ariadne-like protein 8 (ARI8) negatively regulates barley stripe mosaic virus (BSMV) infection. A) Transient overexpression of ARI8 inhibits BSMV infection. BSMV was co-expressed with ARI8 or an empty vector (EV, control) via agroinfiltration. Leaf tissues were collected at 48 h post-infiltration (hpi) and analyzed by immunoblotting. B) Quantification of the triple gene block 1 (TGB1) protein accumulation shown in (A). C) Silencing of *ARI8* in *Nicotiana benthamiana* via tobacco rattle virus (TRV)-induced gene silencing enhances BSMV infection. *N*. *benthamiana* plants infected with TRV–EV or TRV–ARI8 were inoculated with BSMV_γb-3×Flag_ at 14 days post-TRV infection. Leaf tissues were collected at 48 hpi for immunoblot analysis. D) Quantification of TGB1 accumulation shown in (C). E) Quantitative reverse transcription–polymerase chain reaction analysis to validate the downregulation of *ARI8* in TRV–ARI8-infected *N. benthamiana* plants. F) Overexpression of *ARI8* had no pronounced effect on BSMV replication. A movement-deficient BSMV construct (RNAα + RNAγ) was agroinfiltrated into *ARI8*-*OE* plants. Infiltrated tissues were harvested at 48 hpi and analyzed by immunoblotting using anti-γb antibodies. G) Quantification of BSMV γb accumulation shown in (F). (H–I) Knockout of *ARI8* had no prominent effect on BSMV replication. In (B), (D), (E), (G), and (I), error bars represent mean ± standard deviation (*n* = 3 or 4 biological replicates). Asterisks indicate statistically significant differences (unpaired two-tailed *t*-test; n.s. = not significant, *P* >0.05; and **P* <0.05).


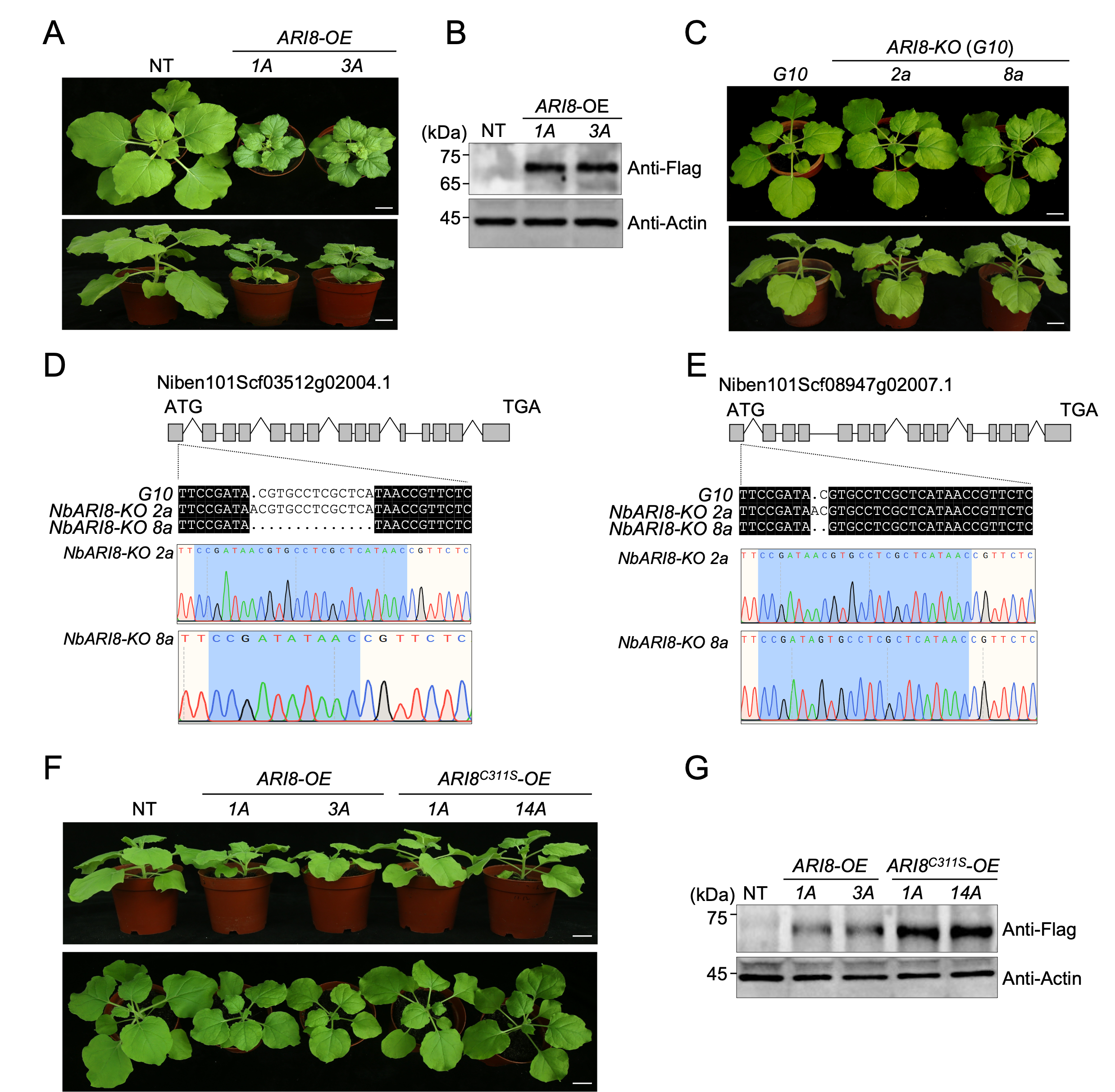


**Figure S7.** Generation of wild-type (WT) Ariadne-like protein 8 overexpressing (*ARI8*-*OE*) and mutant ARI8 overexpressing (*ARI8^C311S^*-*OE*) plants in the WT *Nicotiana benthamiana* background and ARI8 knockout (*ARI8-KO*) plants in the *G10* background. A) Phenotypes of non-transgenic (NT) and *ARI8-OE* *N. benthamiana* plants at 32 days after seeding. Scale bar = 3.5 cm. B) Immunoblot analysis confirming ARI8 protein expression in *ARI8-OE* plants. C) Phenotypes of *G10* and *ARI8-KO* *N. benthamiana* plants at 30 days after seeding. Scale bar = 3.5 cm. D–E) Target site of the single guide RNA (sgRNA) within the genomic fragment of ARI8 or its isoform. ARI8 and homologous amino acid sequences were obtained by BLAST searches against the Solanaceae Genomics Network (<https://solgenomics.net/tools/blast/>). Exons are shown as gray boxes, and introns are shown as black lines. The sgRNA target site was designed within the coding region of the first exon based on genomic sequence analysis. Base insertions and deletions were identified in two independent *ARI8-KO* T1 lines. F) Phenotypes of NT, *ARI8-OE*, and *ARI8^C311S^-OE* *N. benthamiana* plants at 28 days after seeding. Scale bar = 3.5 cm. G) Immunoblot analysis confirming the expression of ARI8 and ARI8^C311S^ proteins in *ARI8-OE* and *ARI8^C311S^-OE* plants using anti-Flag antibody. Actin was used as the loading control.


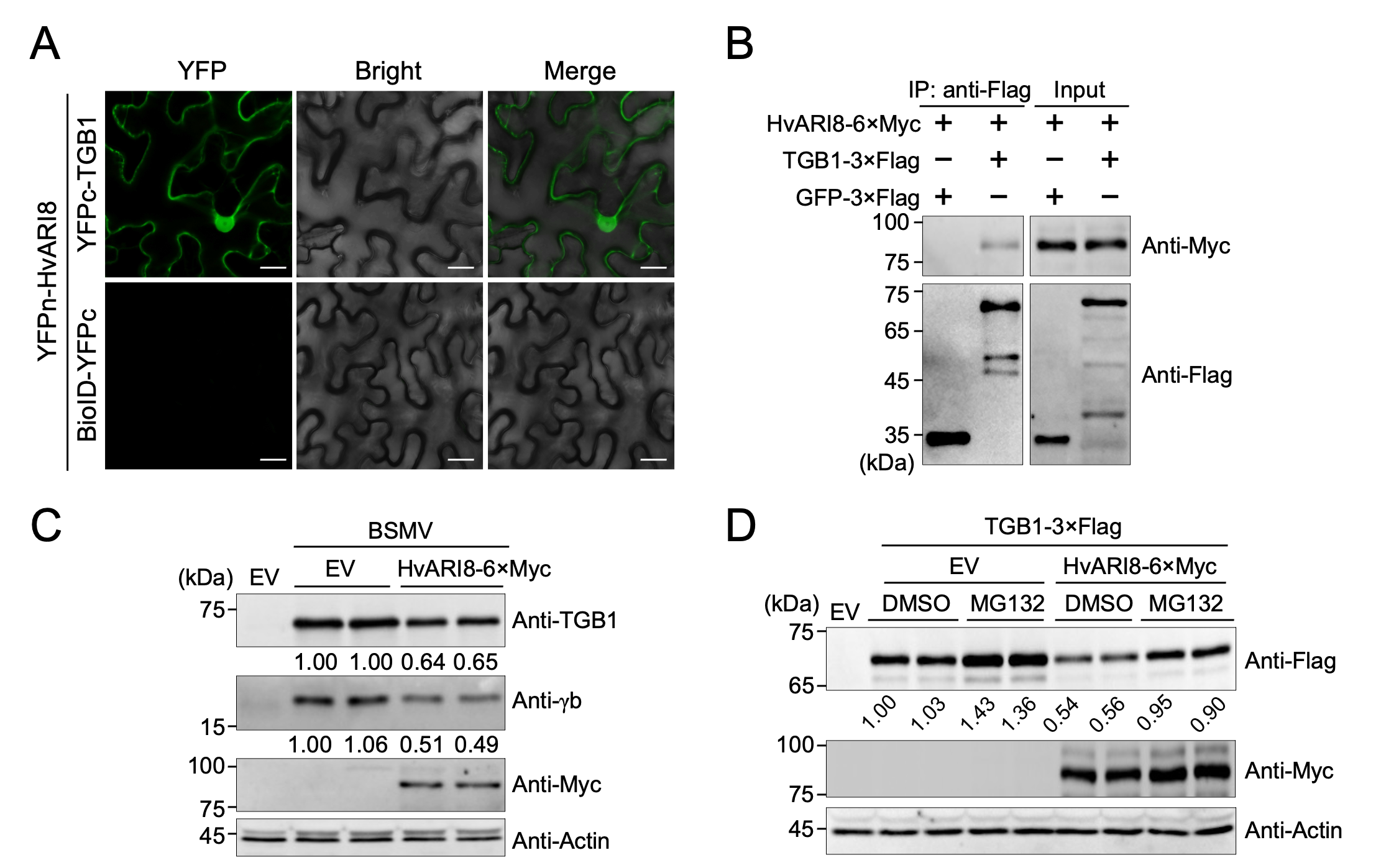


**Figure S8.** *Hordeum vulgare* Ariadne-like protein 8 (*Hv*ARI8) interacts with the triple gene block 1 (TGB1) protein and promotes its degradation via the 26S proteasome. A) Bimolecular fluorescence complementation (BiFC) assay showing the interaction between *Hv*ARI8 and TGB1. *Hv*ARI8 fused with the N-terminal fragment of yellow fluorescent protein (YFPn–*Hv*ARI8) was co-infiltrated with TGB1 fused with the C-terminal fragment of YFP (YFPc–TGB1) or BioID–YFPc into *Nicotiana benthamiana* leaves. Reconstituted YFP fluorescence signals were observed using confocal microscopy. Scale bars represent 20 µm. B) Co-immunoprecipitation (Co-IP) analysis confirming the interaction between *Hv*ARI8 and TGB1. C) Transient overexpression of *Hv*ARI8 suppresses barley stripe mosaic virus (BSMV) infection. D) Overexpression of *Hv*ARI8 decreases TGB1 protein accumulation, a process that is alleviated by MG132 treatment. TGB1–3×Flag was co-expressed with *Hv*ARI8 or an empty vector (EV, control) in *N. benthamiana* leaves. At 36 h post-infiltration (hpi), 100 µM MG132 or an equal volume of dimethyl sulfoxide (DMSO, control) was infiltrated into the previously infiltrated regions. Leaves were harvested 12 h later for immunoblot analysis. Actin was used as the loading control.

**
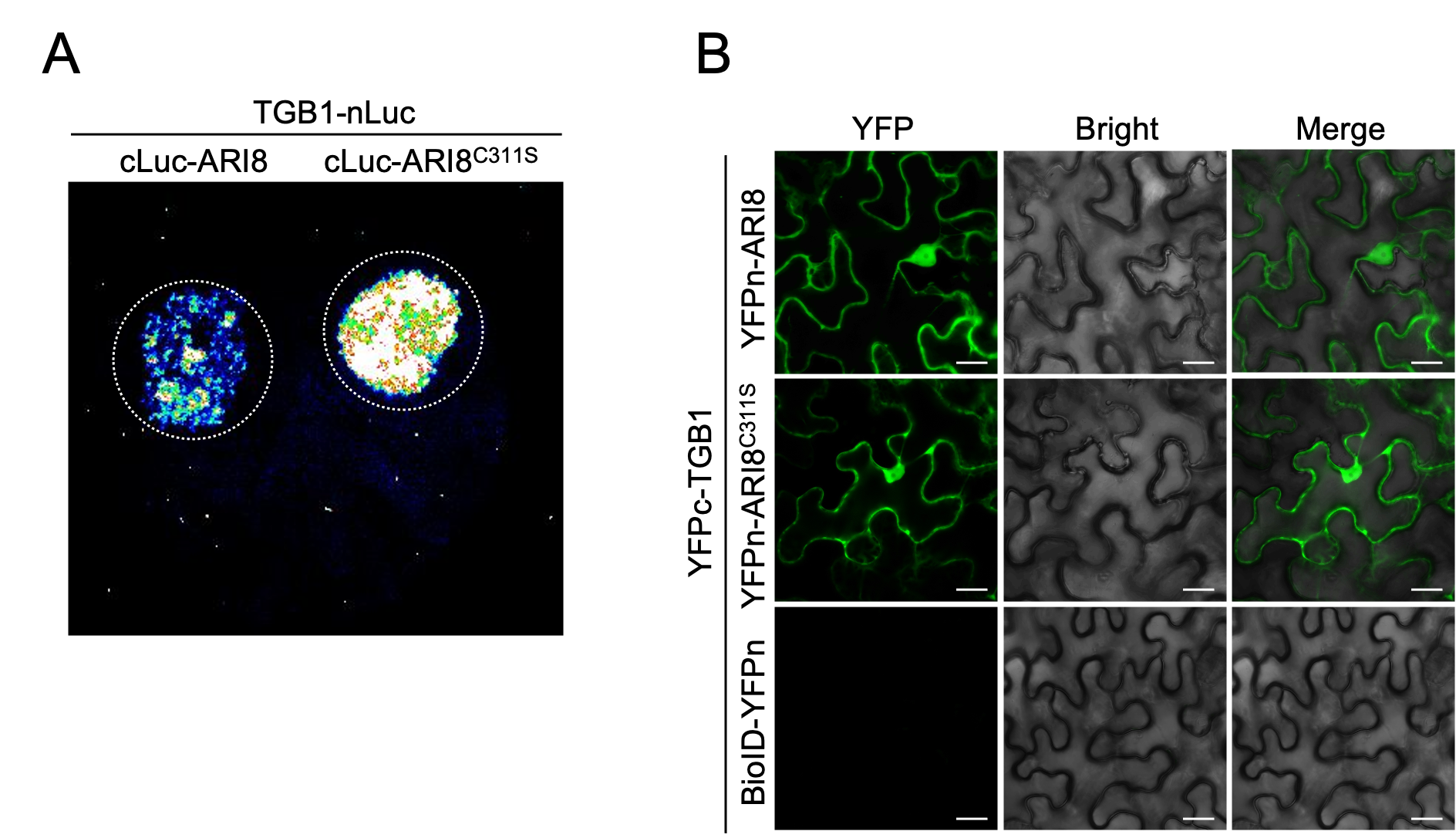
 Figure S9.** Luciferase complementation imaging (LCI) (A) and bimolecular fluorescence complementation (BiFC) (B) assays showed that the mutant Ariadne-like protein 8 (ARI8^C311S^) retained its ability to interact with the triple gene block 1 (TGB1) protein.

**Table S1.** List of candidate proteins potentially interacting with the triple gene block 1 (TGB1) protein of barley stripe mosaic virus (BSMV) and identified by co-immunoprecipitation (Co-IP) coupled with mass spectrometry analysis. Please see the attached Zip file.

**Table S2.** List of primers used in this study.

| **Plasmids** | **Forward Primer (5'-3')** | **Reverse Primers (5'-3')** |
| --- | --- | --- |
| YFPn-ARI8 | AGGCCTACTAGTGGATCCGTCATGGCATCGGAGGATGAATTGATGATG | GGGAGCGGTACCCTCGAGGTCTCAACGGCGCTGCTGGCACATC |
| YFPc-TGB1 | AGGCCTACTAGTGGATCCGTCATGGACATGACGAAAACTGTTGAGG | GGGAGCGGTACCCTCGAGGTCTCATTTGGCCTTGAACCAACTGTGGTC |
| GFP-ARI8 | GACTCAGATCTCGAGCTCAAATGGCATCGGAGGATGAATTGATGATG | GACTGCAGAATTCGAAGCTTTCAACGGCGCTGCTGGCACATC |
| ARI8-3×Flag / ARI8^C311S^-3×Flag | GCCTGGCGCGCCACTAGTGATGGATGGTTCTGGTCAGCAGTCGG | GTCGACAGTACTATCGATGGACATGTGCTGGTATTCAGGATTAA |
| 6×His -ARI8^C311S^ | GAGAAAAATCAAGGGAGCATGCATATCACGTGCACGCCACCCTG | GCACGTGATATGCATTCGCCCTTGATTTTTCTCAATTGGCCGCT |
| TRV-ARI8 | CGCGGATCCATTATAGAGGCCTGGTTGCAGATAG | GCTCTAGAGAGACCATTTTCTAAGGCTTGGACC |
| 6×His -ARI8 | GGACAGCCCAGATCTGGGTACCATGGCATCGGAGGATGAATTGATG | GTGGTGGTGGTGGTGCTCGAGTCAACGGCGCTGCTGGCAC |
| ARI8-nLUC | GGACGAGCTCGGTACCCGGGATCCATGGCATCGGAGGATGAATTGATGATG | CCCGGGACGCGTACGAGATCTGGTCGACACGGCGCTGCTGGCACATC |
| cLUC-ARI8^C311S^ | GTCCCGGGGCGGTACCCGGGATCCCATGGCATCGGAGGATGAATTGATGATG | TACGAACGAAAGCTCTGCAGGTCGACTCAACGGCGCTGCTGGCACATC |
| cLUC-TGB1 | GTCCCGGGGCGGTACCCGGGATCCCATGGACATGACGAAAACTGTTGAGG | TACGAACGAAAGCTCTGCAGGTCGACTTATTTGGCCTTGAACCAACTGTGG |
| ARI8-mCherry | CTCGAGCTCAAGCTTGTCGACATGGCATCGGAGGATGAATTGATGATG | CATTCCTCCGCCGGGCCCGTCGACGGCGCTGCTGGCACATC |
| pKSE401-KO-ARI8 | AATAATGGTCTCTATTGGTTATGAGCGAGGCACGTATGTTTTAGAGCTAGAAATAGC | ATTATTGGTCTCTAAACCTGGTTTGCAGATGAAGGAACAATCTCTTAGTCGACTCTAC |
| pMAL-MBP-TGB1-HA / pCDFDuet-MBP-TGB1-HA-UBA1-S | AGGGAAGGATTTCAGAATTCATGGACATGACGAAAACTGTTGAGG | ACGTCGTATGGGTAAGGCCTTTTGGCCTTGAACCAACTGTGG |
| pACYCDuet-ARI8-Myc / pACYCDuet-ARI8-Myc-UBC8-S | CATCACCAAGCCAGGGATCCATGGCATCGGAGGATGAATTGATGATG | ATGAGCTTCTGCTCAGGCCTACGGCGCTGCTGGCACATC |
| AIR8-6×Myc | CTTCGAATTCTGCAGTCGACATGGCATCGGAGGATGAATTGATGATG | CTTTTGTTCACCTCCGCCGGATCCACGGCGCTGCTGGCACATC |
| YFPc -TGB1^PVX^ | AGGCCTACTAGTGGATCCGTCATGGATATTCTCATCAGTAGTTTGAAAAGTTTAGG | GGGAGCGGTACCCTCGAGGTCCTATGGCCCTGCGCGGACATATGTC |
| YFPc -TGB1^BNYVV^ | AGGCCTACTAGTGGATCCGTCATGGTCCAAGTACAGCGTAGAACGG | GGGAGCGGTACCCTCGAGGTCTTATCTATCTTCGCAAAAGGTATCTCCGGTAC |
| YFPn-HvARI8 | AGGCCTACTAGTGGATCCGTCATGGACTCCGAGGACGACATGC | GGGAGCGGTACCCTCGAGGTCCTATCGGTGTTGGTGGTCACAAGC |
| HvARI8-6×Myc | CTTCGAATTCTGCAGTCGACATGGACTCCGAGGACGACATGC | CTTTTGTTCACCTCCGCCGGATCCTCGGTGTTGGTGGTCACAAGC |
